# Consensus driven by a minority in heterogenous groups of the cockroach *Periplaneta americana*

**DOI:** 10.1016/j.isci.2021.102723

**Published:** 2021-06-12

**Authors:** Mariano Calvo Martín, Max Eeckhout, Jean-Louis Deneubourg, Stamatios C. Nicolis

**Affiliations:** 1 Center for Nonlinear Phenomena and Complex Systems (Cenoli), Université libre de Bruxelles, Campus Plaine, Boulevard du Triomphe 155, 1050 Brussels, Belgium; 2 Evolutionary Biology and Ecology, Department de Biologie des Organismes, Université libre de Bruxelles, Campus Solbosch, Avenue Franklin Roosevelt 50, 1050 Brussels, Belgium

**Keywords:** Biological Sciences, Ecology, Ethology, Social behaviour

## Abstract

Many social species are able to perform collective decisions and reach consensus. However, how the interplay between social interactions, the diversity of preferences among the group members and the group size affects these dynamics is usually overlooked. The collective choice between odourous and odorless shelters is tested for the following three groups of social cockroaches (*Periplaneta americana*) which are solitary foragers: naive (individuals preferring the odorous shelter), conditioned (individuals without preference), and mixed (combining, unevenly, conditioned, and naive individuals). The robustness of the consensus is not affected by the naive individuals' proportion, but the rate and the frequency of selection of the odorous shelter are correlated to this proportion. In mixed groups, the naive individuals act as influencers. Simulations based on the mechanisms highlighted in our experiments predict that the consensus emerges only for intermediate group sizes. The universality of these mechanisms suggests that such phenomena are widely present in social systems.

## Highlights

• Consensus is group size dependent
• Consensus is not affected by an aversive group conditioning
• Consensus is robust regardless the diversity of individual preferences in a group
• Group choice is controlled by the preference of a minority of individuals

## Graphical abstract

**Figure:**
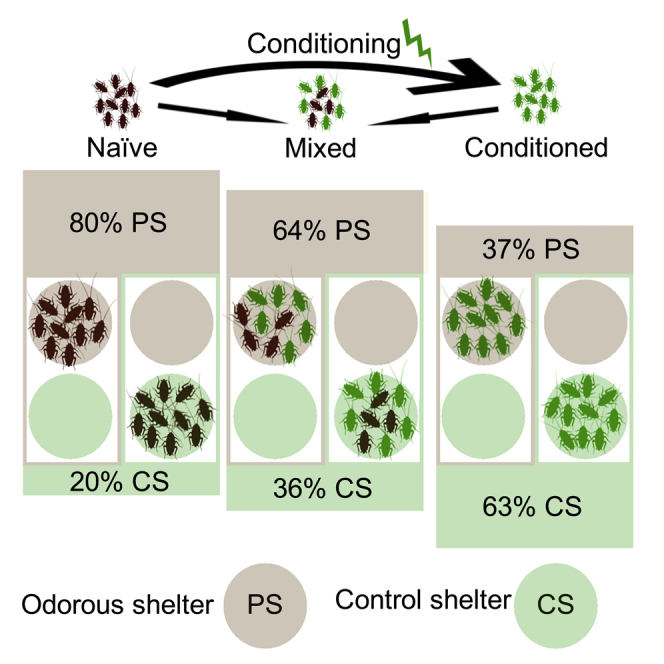

## Introduction

Aggregation, which results in an uneven distribution of individuals through space, is a widespread behavior occurring in living organisms, from unicellular organisms to mammals (Camazine et al., 2003; Sumpter, 2010). In arthropods, this behavior can be observed at specific life cycle stages (e.g., during larval states) (Krafft et al., 1986), under unfavourable external conditions (e.g., winter) (Prokopy and Roitberg, 2001) or, in the case of gregarious and eusocial species, in numerous daily activities (Costa, 2006; Jeanson et al., 2004). In this process of aggregation governed by collective decision-making, organisms take decisions to join or to leave an aggregate on the basis of personal information and information provided by conspecifics (public information).

During decision-making, animals respond to biotic (i.e., conspecifics, predators, odors) and abiotic (i.e., temperature, light/colors, humidity) cues to guide themselves through the environment (Cardé and Willis, 2008; Steinbrecht, 2007). The strength of the response depends not only on the intensity or the concentration of the cues (Cloudsley-Thompson and Constantinou, 1987; Strutz et al., 2014) but also on the state of the individual (hunger, life cycle) (Robinson, 1985; Verhoef and Witteveen, 1980) and is therefore context dependent (Endres and Fendt, 2007; Philippe et al., 2016; Plenzich and Despland, 2018). In the case of arthropods, one of the most important factors is the chemical environment (for simplicity, hereafter, odors) (Koehl, 2006; Zimer-Faust et al., 1985). Different receptors, mainly present in the antenna, generate a response depending on the type of odor and its concentration (Bell and Adiyodi, 1981; Paoli et al., 2020).

The outcome of collective decisions, in response to the environmental cues and to the conspecifics, modifies the structure of the group in space (e.g., migration of locusts) and in time (e.g., aggregation of beetles during winter) (Copp, 1983). Still, these decisions are subjected to variabilities not only linked to the environment but also to those among group members, resulting in different behavioral outcomes for the same quality resource. These interindividual variabilities can be phenotypical with or without a morphological expression and constant through the life of the individual (i.e., gene, sexual, size) (Eckholm et al., 2011; Krause et al., 1996; Nicolis et al., 2020), but they can also be temporary (i.e., desiccation, hormonal, age) (Krafft et al., 1986; Robinson, 1985) or acquired (i.e., life history: memory, social learning) (Battesti et al., 2015; Ravary et al., 2007). These variabilities are not mutually exclusive and can be at the origin of the variability of preferences. The extreme case of such interindividual variability is the division of labor in eusocial animals (Jeanson et al., 2008; Weidenmuller, 2004).

Collective decisions wherein group members cannot be coerced into their decisions by others are part of many social animals, ranging from (eu)social arthropods to mammals. In these groups, individuals influencing each other may have different behaviors or preferences. In such heterogeneous groups this influence can, lead to social learning (Laland, 2004; Mathis et al., 1996; Scheid and Noë, 2010). Moreover, these individual behaviors can be associated to other cues (Croney and Newberry, 2007; Haney and Lukowiak, 2001). A common case concerns groups where individuals having the same preference but different response thresholds or different response intensities are able to perform collective decisions through amplification of individual preferences (Planas-Sitjà et al., 2015). However, individual preferences can be opposite, and therefore, two collective decisions outcomes can be observed within a group. A first one is a fission, resulting from individuals having very different preferences and/or differential association between individuals (e.g., kinship, social status) (Carter et al., 2013; Kerth and König, 1999). The second outcome, being the group's cohesion (e.g., aggregation in a unique site), resulting from rather not so different preferences and/or a strong interattractions leading (in some cases) to a consensus.

In democratic groups, the consensus is reached by choosing the most frequent preference of the group members (Conradt and Roper, 2007). In nondemocratic groups, where some individuals (leaders) influence more than others, a consensus is still reached and the group's decision shifts toward the preference of these individuals (Conradt and Roper, 2005; Petit and Bon, 2010; Webster and Ward, 2011). Furthermore, when considering the emergence of consensus associated with the aggregation, two extreme cases can be considered. In the first one, the individuals are in contact (e.g., visually, physically) and they coordinate their movements (e.g., school, flock, queue) (Bourjade et al., 2009; Fitzgerald and Pescador-Rubio, 2002; Rosenthal et al., 2015; Strandburg-Peshkin et al., 2013; Ward and Webster, 2016). In this case, the collective choice of the aggregation site precedes the synchronized settlement of the individuals on this site. In the second case, the animals individually explore the environment before joining the site. The settlement of individuals appears to be desynchronized, but it is influenced by the conspecifics present on the site (many gregarious arthropods) (Bell et al., 2007; Costa, 2006; Lazzari and Lorenzo, 2009). In this situation where interindividual influences are discontinuous, the emergence of a consensus is less intuitive and particularly in heterogeneous groups, which is the main focus of the present study.

In a binary choice experiments with groups of 10 nymphs (L6-L7 instar) of the American cockroach (*Periplaneta americana*), we test the capacity of naive, conditioned (classical aversive conditioning), and mixed groups (a combination of conditioned individuals and a minority of naive individuals) to reach a consensus during settlement. The insects, like many others, individually explore the environment and choose to settle between one shelter without odor (hereafter CS) and one with peanut butter odor (hereafter PS) (see Figure S10 and STAR methods). Cockroaches show a spontaneous preference for some odors like that of peanut butter (Nalyanya and Schal, 2001), the attractive molecule being the 1-hexanol (Karimifar et al., 2011). Indeed, it is most likely that 1-hexanol, a volatile component of peanut butter, is an indicator of lipids or lipids degradation source (Feussner and Wasternack, 2002) and therefore an attractant molecule. The conditioned individuals have lost their preference for this odor or developed an aversion for it. Cockroaches are aversively conditioned (electric shocks associated to a peanut butter scented shelter) in group of 10 (see Figures S8 and S9 and STAR methods). The hypotheses raised here are that regardless of the individual information and behavior, a consensus is always achieved and that group preference (consensus) and group dynamics are regulated by the group composition. In other words, consensus will always be reached, but the frequency of selection of the shelters (with or without odor) will depend on the composition of the group. These hypotheses are supported by the literature regarding the strong interattraction between the cockroaches (Leoncini and Rivault, 2005; Lihoreau et al., 2012) and their capacity to reach a consensus. Furthermore, we develop a mathematical model based on the performed experiments and in agreement with the literature. It suggests that the conditioning does not affect the interattractions but affects only individual preferences. Finally, the adaptive value of these phenomena is discussed.

## Results

### Global dynamics

**Figure 1:**
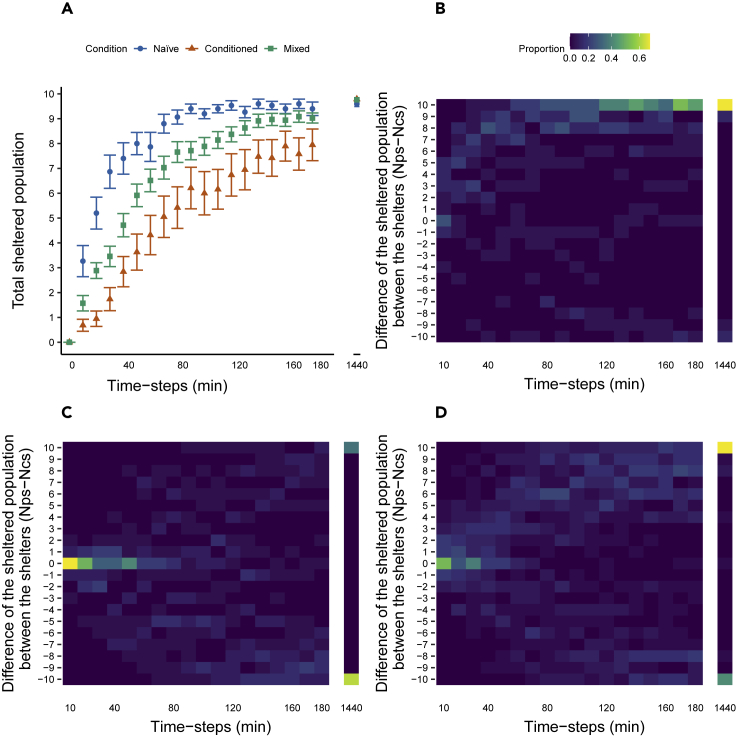
Evolution of the population over times Sheltered population of the experiments. (A) Mean ± SEM of the total sheltered population over time (minutes) for the naive (blue), the conditioned (orange) and the mixed (green) conditions. (B–D) Heatmap (frequency) of the difference of the sheltered population between the PS and the CS (N_ps_ - N_cs_) every ten minutes. (B) naive condition. (C) Conditioned condition. (D) Mixed condition.

For all three conditions, groups are composed of 10 nymphs of *P. americana*. Conditions differed on the ratio of naive/conditioned individuals: for the naive condition is 10/0 (N_trials_ = 15); for the conditioned condition is 0/10 (N_trials_ = 19); and for the mixed condition is 4/6 (N_trials_ = 35) for more details see STAR methods. In the three conditions, the total sheltered population increases over time (Figure 1A). The population of naive individuals shelters faster than those of mixed condition, and the conditioned groups shelter even slower: during the first three hours of the experiment, the naive condition has a higher total sheltered population, followed by the mixed and last the conditioned. At 3 h, the sheltered populations (mean ± sd) are naive: 9.4 ± 1.1; mixed: 9.0 ± 1.2; conditioned: 7.9 ± 2.8. A resampling test shows significant differences (resampling test: p < 0.05) between 10 and 150 min (naive *vs* mixed) and between 10 and 180 min (naive *vs* conditioned and conditioned *vs* mixed) (Figure S1). After 24 h, the differences between conditions are negligible, the mean ± sd percentage of sheltered individuals being larger than 98% for all conditions (naive: 9.8 ± 0.4; conditioned: 10 ± 0; mixed: 9.97 ± 0.17; resampling test: p > 0.9). In the mixed case, a permutation test shows that the proportion of the total sheltered naive individuals is significantly larger (permutation test: p < 0.05) than the one of the conditioned individuals between 20 and 180 min. However, at 24 h, the proportions of sheltered naive and conditioned individuals are equal, and their means are greater than 0.99.

### Collective choice and consensus

The comparison between the binomial distribution and the experimental data show that positive interactions between individuals are at work (observed) after 10 min for naive and mixed condition and after 30 min for conditioned condition (permutation test: p < 0.05, for details on the test, see STAR methods). The rapid emergence of a strong asymmetry, during the experiment, reflects the selection of a particular shelter. Indeed, Figures 1B–1D show the difference between the population of the shelters (N_ps_ - N_cs_) over time, where N_ps_ (N_ps_) is the number of individuals in the PS(CS). Bimodal distributions of the difference between the sheltered populations (N_ps_ - N_cs_) are observed from particular time steps (80 min for naive, 150 min for conditioned, and 90 min for mixed) until the end of experiments for all conditions (Cramer-von Mises test: Fisher and Marron method; p < 0.05, Table S1). Furthermore, the positions of the modes in these bimodal distributions tend to be symmetrical (Table S1).

Before observing a bimodal distribution there are particular times *T*_*w*_ from which a shelter becomes the most occupied one until the end of the trial (mean ± SD: naive: 15 ± 6.5 min, conditioned: 37.2 ± 38.2 min and mixed: 20.9 ± 17.1 min) (Resampling test: naive vs mixed - p = 0.073; naive vs conditioned - p < 0.001; conditioned vs mixed - p < 0.001). In addition, a survival analysis on the values of *T*_*w*_ shows no significant difference between the selected shelter (PS *vs* CS) for the three conditions (naïve – Peto-Peto test: *χ*^2^_ddl=1_ = 0.1, p = 0.8, Figure S2A; conditioned – Peto-Peto test: *χ*^2^_ddl=1_ = 0.6, P = 0.5, Figure S2B; mixed – Peto-Peto test: *χ*^2^_ddl=1_ = 2.4, p = 0.1, Figure S2C). However, between the conditions, regardless of the selected shelter, there is a significant difference of the value of *T*_*w*_ (Peto-Peto test: *χ*^2^_ddl=1_ = 9.2, p = 0.01, Figure S2D). A pairwise comparison between the conditions show that there is a significant difference between naive and conditioned (p = 0.006, P adjustment method: Hommel) and no significant differences between naive and mixed (P = 0.09, P adjustment method: Hommel) and between mixed and conditioned (p = 0.09, P adjustment method: Hommel).

At 24 h, 97% of the experiments end with 90% or more of the total sheltered population in the same shelter (100% for naive and conditioned, 94% for mixed). This shows that the level of consensus (defined here as the majority of the individuals that share the same decision and that no other group is formed elsewhere) is independent of the shelter type. These collective choices can actually be predicted at 180 min, as the population inside the selected shelter at 180 min is positively correlated to the one at 24 h (Pearson correlation test: naive – *ρ* = 0.93, p < 0.0001; conditioned – *ρ* = 0.87, p < 0.0001; mixed – *ρ* = 0.92, p < 0.0001; mixed conditioned individuals - *ρ* = 0.92, p < 0.0001; mixed naive individuals - *ρ* = 0.86, p < 0.0001). This correlation suggests an absence of complete deconditioning of the conditioned individuals (for the conditioned and the mixed condition) during the trials.

### Shelter preference and mechanisms

At 24 h, the proportion of trials that ended with 90% or more of the total sheltered population within the PS(CS) is 0.8(0.2) for the naive, 0.37(0.63) for the conditioned, and 0.64(0.36) for the mixed (Table 1). In this last condition, only 2 trials of the 35 ended with individuals distributed among both shelters (7 vs 3 and 5 vs 5 PS vs CS, respectively). The population under the PS was more frequently higher than CS with a significant difference for the naive at every time step (permutation test: p < 0.05). For the mixed, as well, the PS is preferred, significantly between 50 min and 180 min (permutation test: p < 0.05) and with a tendency at 24 h, (permutation test: p = 0.056). However, for the conditioned, the population under the CS was more frequently higher than under the PS, nonetheless, no significant difference is observed at any time-step (Permutation test: p > 0.05). The 3 conditions, naive; mixed; conditioned, differ on the proportion of conditioned individuals (0, 0.6 and 1). The condition has an influence on the shelter selection which is independent of the time expended in the experiment (Figure S3, linear regression – naive: F = 0.08; p = 0.8; R^2^ = 0.003 – conditioned: F = 0.5; p = 0.8; R^2^ = 0.003 – mixed: F = 0.005; p = 0.9; R^2^ = 0.001). Nonetheless, the proportion in the PS between conditions is significantly different (value at the origin of the linear regression – naive estimated = 0.78 ± 0.02; *t =* 33.44; p < 0.001 – conditioned estimated = 0.4 ± 0.03; *t* = 13.34; p < 0.001 – mixed estimated = 0.63 ± 0.02; *t =* 33.98; p < 0.001, Table S2 and). Furthermore, as suspected, the influence of conditioned individuals on the population in the PS follows a logistic distribution:

(Equation 1) $$P_{ps}=\frac{e^{\left(\alpha -\beta N_{conditioned}\right)}}{1+e^{\left(\alpha -\beta N_{conditioned}\right)}}$$

where *P*_*ps*_ is the proportion of sheltered individuals in the PS, *α* determines the basal proportion of individuals sheltering in the PS, and β is the influence of the number of conditioned cockroaches (*N*_*conditioned*_). A nonlinear least squares fit of Equation 1 shows a significant fit of the parameters at every time step (see Table S3 and Figure S4 for the fittings of the parameter and significances).

**Table 1:** Summary result for each condition

	Naive		Conditioned		Mixed	
	N = 15		N = 19		N = 35	
	PS	CS	PS	CS	PS	CS
Mean ± sd sheltered individuals at 3 hr	7.5 ± 3.5	1.9 ± 3.2	2.9 ± 3.8	5.0 ± 4.4	5.6 ± 3.7	3.5 ± 3.9
Mean ± sd sheltered individuals at 24 hr	7.9 ± 4.1	1.9 ± 4.0	3.7 ± 4.9	6.3 ± 4.9	7.3 ± 4.6	3.7 ± 4.7

Population inside the shelter at 3 and 24 hr for the naïve (10 naïves individuals), the conditioned (10 conditioned individuals) and the mixed (4 naïves and 6 conditioned individuals) conditions.

### Mixed condition

In the mixed condition, groups are composed of 4 naive and 6 conditioned individuals marked with red and black dots, respectively, on their pronotum (see experimental procedure in the STAR methods). Ninety-four percent of trials ended up with 9 or 10 individuals of the group resting in one shelter. A *χ*^2^ test shows that during the experiment, the fraction of naive sheltered individuals is larger than the fraction of conditioned ones, from 30 to 170 min of the trials. Moreover, at each time step (except at t = 20 min), the fraction of naive sheltered individuals is linearly correlated to the fraction of conditioned sheltered individuals (Pearson correlation, p < 0.05).

### Mechanism of shelter selection and model

The sheltering dynamics result from the entries to the shelters and the exits from them, which depend on the individual probabilities of joining and leaving the shelters. Usually, these probabilities depend on the qualities of this shelter and its housed individuals. Based on previous works (Ame et al., 2006; Calvo Martín et al., 2019), the probability to join a shelter at every time step is independent of the sheltered population or weakly influenced by it. However, the individuals respond to the chemical qualities of the site (odor, humidity), and thus, the joining probability can vary if the two shelters are different. The simplest expression of the individual joining probability to the PS or the CS is as follows:

(Equation 2) $$J_{ps}=\theta \mu_{ps};J_{cs}=\theta \mu_{cs}$$

where θ is the individual global probability to find a shelter while exploring by unit of time (or how fast the animal explores), the larger θ is, the faster the growth of the sheltered population will be. As for *μ*_*ps*_*(μ*_*c*_*),* it is the relative individual response to the quality of the shelter PS (CS). As for the leaving probability at every time step, it depends on the shelter quality as well as on its population. Like many other gregarious animals, cockroaches are retained by conspecifics and the probability of leaving decreases with the sheltered population (Laurent Salazar et al., 2017). Here, we assume that this probability exponentially decreases with this population (Calvo Martín et al., 2019):

(Equation 3) $$Q_{ps}=\rho_{ps}e^{-\varsigma_{ps}\left(X_{ps}-1\right)};\rho_{cs}e^{-\varsigma_{cs}\left(X_{cs}-1\right)}$$

where *ρ*_*ps*_*(ρ*_*cs*_*)* is the leaving probability when individuals are isolated by unit of time (thus depending on the shelter quality), ς_*ps*_*(*ς_*cs*_*)* is the social strength, and *X*_*ps*_ (*X*_*cs*_) is the number of individuals present in the shelter.

The values of *μ*_*ps*_*, μ*_*cs*_ in Equation (2) depend on the type of cockroaches. It has been previously shown that the odor of peanut butter is attractive for naive individuals (Karimifar et al., 2011), and therefore, we can consider that for these individuals *μ*_*ps*_ *> μ*_*cs*_. As for the conditioned individuals, the values of *μ*_*ps*_ and *μ*_*cs*_ are either reversed or they become equal, as the attraction toward the odor of peanut butter has been inhibited. The same conclusions can *a priori* be drawn for the parameter *ρ,* but no data suggest that this odor has a retention effect. Therefore, the minimal assumption retained here will be that *ρ*_*ps*_ *= ρ*_*cs*_ *=ρ*.

Previous works show that sociality was affected negatively in an attractive shelter (Calvo Martín et al., 2019; Günzel et al., 2021; Laurent Salazar et al., 2017). This is not observed in our experiments as the level of consensus is greater than 0.9 whatever the type of shelter and of individuals. Indeed, to observe such uneven bimodal distribution at the end of the experiments, the social strength has to be strong. We therefore assume that the social strength parameter ς of the naive and conditioned individuals are equal.

A first approach to approximate the values of θ is to neglect the exits from the shelters (*ρ* = 0). The fitting of the total sheltered population with such simplification gives the minimal value of θ (see Table 2 and supplemental information). Moreover, to approximate the parameter values (θ *μ*, *ρ,* and ς) and identify their extremes values (Table 2) that are compatible with the experimental result at 24 h, we numerically integrated the master equation formulation (Nicolis and Prigogine, 1977) of our model which gives the probabilities of observing *i* individuals in PS and *j* in CS (for both the naive and the conditioned individuals, see supplemental information). These extreme theoretical values can be considered as a confidence interval. This first analysis shows that for the two conditions (naive and conditioned), the parameter values (*ρ* and ς) are in the same ranges of values, but not θ and *μ*. Although this analysis is compatible with the experimental results at 24 h, it is only partially consistent with the full dynamics.

**Table 2:** Estimated limits of the parameter of the model

	Naive		Conditioned	
	Min	Max	Min	Max
ϴ	0.0007/0.0006^a^	0.005	0.0003/0.0001^a^	0.005
μ_ps_	0.55	0.7	0.4	0.45
ρ	0.001	0.0025	0.0015	0.0025
ς	0.9	1.4	1.0	1.3

Theoretical approximations of the parameter of the model at 24 hr.

a Estimated by a fitting of the total sheltered population. See section Mechanism of shelter selection and model in the supplemental information.

To further approximate the parameters of the model (θ, *μ*, *ρ,* and ς), we performed dynamical stochastic simulations of the model (10,000 realizations). We summarize hereafter the different steps of the simulations:

• At *t* = 0, all individuals are outside.
• At each time step (second), individuals outside the shelters have a probability *J*_*ps*_ or *J*_*cs*_ (Equation 2) to find and join the PS or the CS.
• At each time step, individuals inside a shelter can leave it with a probability *Q*_*ps*_ or *Q*_*cs*_ (Equation 3).
• The simulations are performed for 10,800 time steps (3 h) and for 10,000 realizations.

**Figure 2:**
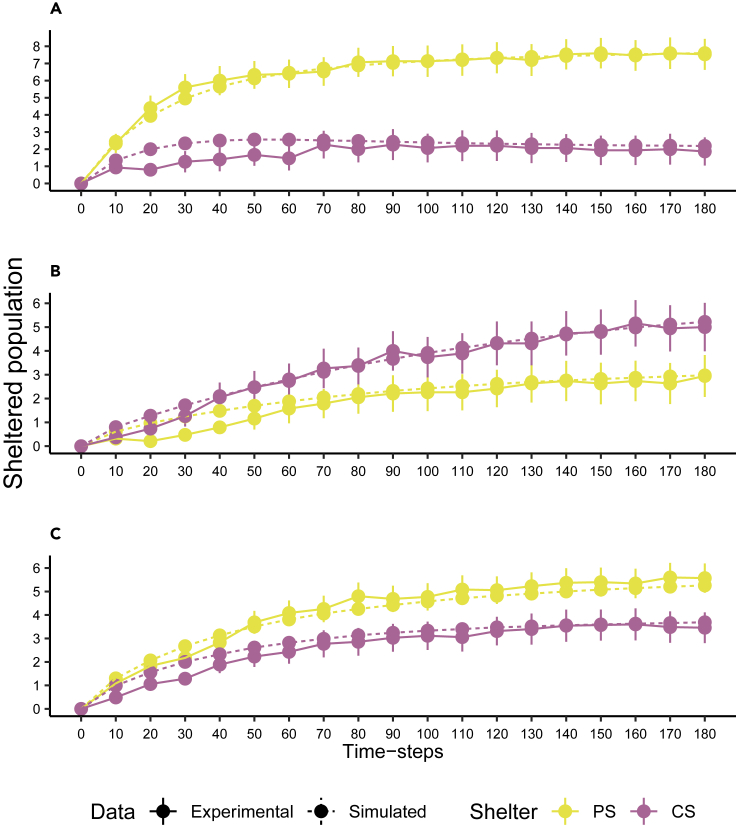
Experimental and simulated dynamic of the sheltered population Mean ± SEM of the population under the PS (yellow) and the CS (violet) over time (minutes) for the experiments (solid lines) and stochastic simulations (10,000 realizations; dashed lines; from Equations 2 and 3; parameter values: Table 3). (A) naive condition. (B) Conditioned condition. (C) Mixed condition.

The outputs of the simulations are used in a resampling test that compares sets of simulated outcomes to the experimental results (see supplemental information). Figure 2 shows the temporal evolution of the sheltered populations from the simulations with the parameter values that yield the best results from the resampling test at every time step (Table 3). Note that only at times 20, 30, and 40 min for the naive condition and at time 10 min for the mixed condition, the resampling tests show a significant difference (p < 0.05, Figure S5A).

**Table 3:** Retained parameter values

	Type of cockroaches	
	naive	Conditioned
ϴ	1 × 10-3 s-1	3.703 × 10-4 s-1
μ_ps_	0.63	0.445
ρ	0.002 s-1	0.002 s-1
ς	1.19	1.19

Estimated parameter values used for the simulations.

**Figure 3:**
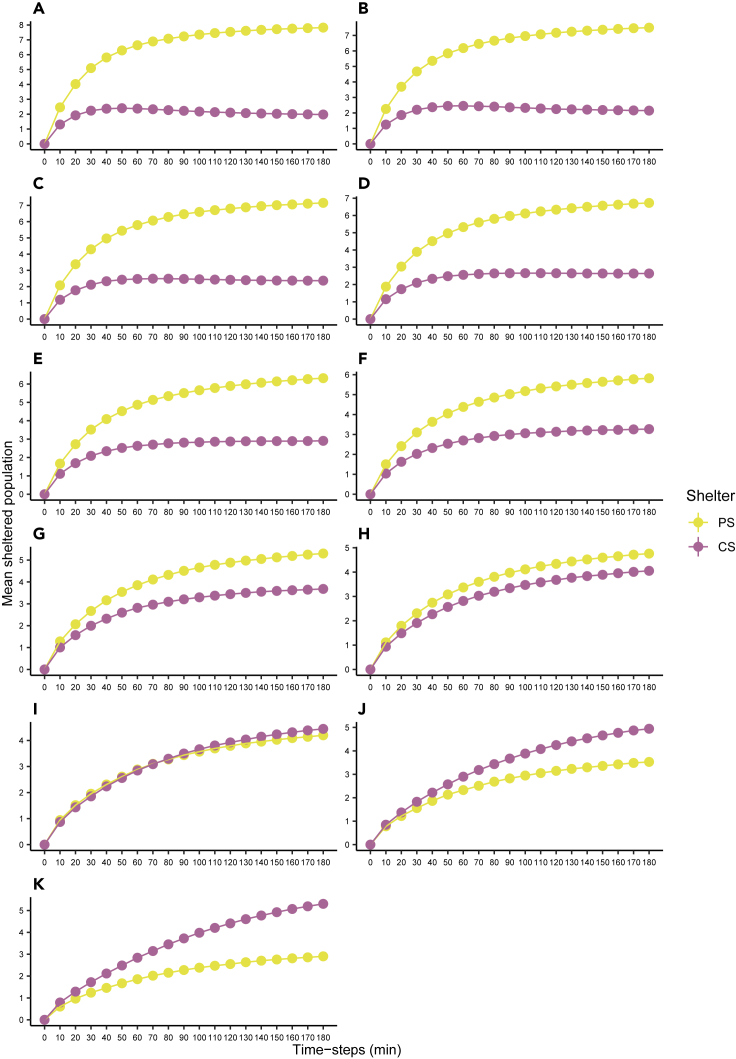
Influence of group diversity Mean simulated (10,000 realizations; stochastic simulations) sheltered population in the PS (yellow) and CS (violet) over time defined by Equations 2 and 3 (parameter values Table 3). (A–K) Different number of naive individuals in mixed groups (N_group_ = 10 individuals): (A) 10. (B) 9. (C) 8. (D) 7. (E) 6. (F) 5. (G) 4. (H) 3. (I) 2. (J) 1. (K) 0.

These results confirm that the naive and the conditioned individuals differ as far as the global probability to find a shelter θ and the response to the odorous shelter *μ*_ps_ are concerned. *A contrario*, the individual-leaving probability *ρ* and the strength of social cohesion ς are the same for both shelters and both types of individuals. Moreover, when the individual responses for the PS (*μ*_ps_) and CS (*μ*_cs_) were kept equal, regardless of the type of individual, the simulated data were significantly different from the experimental data (see Figure S5B). Finally, the model predicts that when the proportion of the conditioned individuals varies from 0 to 1 (Figure 3), a clear selection of the CS by the group will only occur for groups with less than 20% of naive individuals. These simulations also predict, at the end, a bimodal distribution that is a characteristic of a strong sociality (Figure S6).

**Figure 4:**
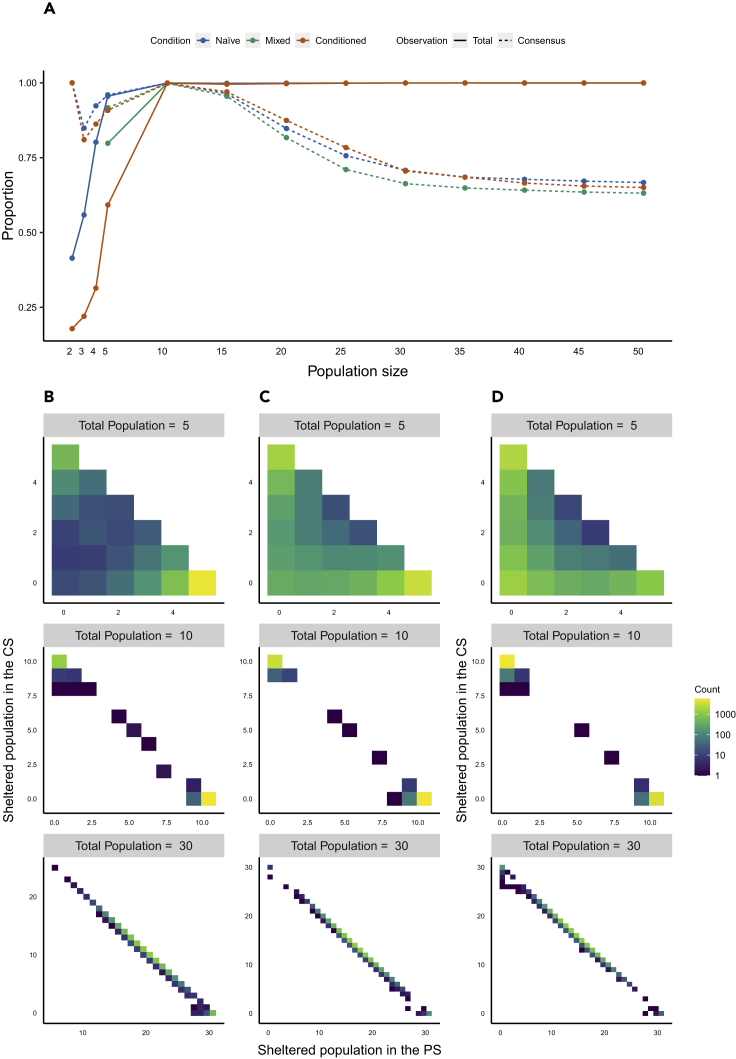
Simulated sheltered distribution Results of stochastic simulations (10,000 realizations) of the model (Equations 2 and 3, parameter values from Table 3) at 24 hr (86,400 time-step). (A) Mean simulated proportion of the total sheltered population (solid line) and of the consensus (dashed line) over total population, keeping the same proportions as in the experimental conditions, naive: 100% of naive individuals (blue); mixed: 40% of naive individuals (green); and conditioned 100% of conditioned individuals (red). (B–D) Two dimensional histograms (10,000 realizations) of the simulated sheltered population in the PS and in the CS for population size of 5; 10; and 30 individuals. (B) naive. (C) Mixed with experimental proportion. (D) Conditioned.

Keeping the experimental proportions of the three groups 100% naive (naive), 40% of naive (mixed), and 0% naive (conditioned), we perform stochastic simulations (10,000 realizations) based on the mechanisms described previously, to study the effect of the population size on the consensus at 24 h. Figure 4A displays the proportion of the total sheltered population and the proportion of simulations ending with a consensus (90% of the population under the same shelter) as a function of the population size for the three conditions. While the proportion of the total sheltered population increases with the population size, the proportion of simulations leading to a consensus exhibits a maximum at a population size between N = 10 and N = 15 individuals. Also note that for the conditioned condition the proportion of simulations leading to a consensus exhibits a minimum at N = 3 owing to their low probability to find a shelter (*ϴ*). Finally, Figures 4B–4D and Figures S7–S9 show the simulated distributions of individuals under the shelters. For low population sizes a non-negligible number of realizations ended up with most individuals outside the shelters, emphasizing the importance of a critical population size in social systems to reach an efficient cooperation. As the population size increases, most of the individuals are sheltered and the system goes from an asymmetrical bimodal distribution (i.e., in each simulation the population ends up being sheltered in one or the other shelter) to a unimodal distribution (i.e., in each simulation the population ends up being distributed among the shelters), the group composition influencing the frequencies of each shelter occupation. The bimodal distribution is the signature of the consensus, and the lack of consensus leads always to a unimodal distribution.

## Discussion

This study sheds light on the mechanisms behind collective decisions and robustness of the consensuses in a model species (*P. americana*). In these cockroaches, like in many other arthropods, the interactions between individuals are linked to specifics activities, in particular, the collective choice of a resting site (Costa, 2006; Duffy and Thiel, 2007). In these settings, we show the importance of the strength of positive social interactions (interattraction) and public information as compared with the diversity of individual preferences. A novel and important result is that consensus is robust independently of the levels of the group heterogeneity. Indeed, almost all trials for the three conditions ended up with at least 90% of the total population being in a same shelter. Another consequence of the cohesiveness of individuals is that, in homogeneous groups, some collective selections are observed in the shelter which do not correspond to the preference of each individual (PS for conditioned, CS for naive) (see also Sumpter (2006)). Nonetheless, naive groups selected more frequently and faster the PS, whereas the conditioned groups selected more frequently the CS. As for the mixed groups, they selected more frequently the PS despite a minority of naive individuals. These results lead to the conclusion that the strength of social interactions remains the same, regardless of the type of individuals (naive, conditioned, mixed). Note that this strength has no reason to be different between the shelters or between types of individuals, as the conditioning of the cockroaches is in group (see conditioning procedure in the STAR methods). However, under higher stress conditions (e.g., food deprivation), the strength of sociability could decrease and a dispersion of a group could be observed, already reported in social spiders and in nomadic caterpillars (Krafft et al., 1986; Plenzich and Despland, 2018).

For the three conditions, most of the experiments (97%) ended up with the selection of a particular shelter (consensus for the PS or the CS) at the third h which is stabilized or is increased further until the 24th h. This suggests that no memory loss occurs during the duration of the experiments, which is in agreement with previous works showing that cockroaches can retain acquired memories up to one week (Balderrama, 1980; Sakura and Mizunami, 2001; Watanabe et al., 2003). However, we do not exclude a weak reversal of the conditioning, when the conditioned individuals visit the PS (Watanabe et al., 2003), and that this reversal could be more marked in the presence of naive individuals in the mixed condition.

The selection of the winning shelter appears early in time, indicating that the process of decision-making is fast and irreversible. The relatively small area of our setup partially contributes to this high rate (Full and Tu, 1991). Nonetheless, the rates of settlement and of consensus are positively correlated to the proportion of naive individuals (Figure 1A) and are therefore impacted by the past events, such as the conditioning of some individuals. In addition, in the mixed groups, the proportion of naive-sheltered individuals is larger than the one of the conditioned-sheltered individuals before reaching a consensus, suggesting that the conditioning procedure not only reduces the preference of the individuals for PS but also their rate of settlement as well. The process of shelter selection being controlled by the individual joining and leaving probabilities, the different sheltering rates highlighted in this article find their origin in the different probabilities of the two types of individuals. While we do not exclude that the probability to join a shelter increases with the sheltered conspecifics (Calvo Martín et al., 2019), in this study, this feedback is neglected. Instead, the naive individuals have a higher probability per time unit (θ) to join a shelter than the conditioned ones, most likely owing to the conditioning method (see Conditioning procedure of the STAR methods). As for the probability of leaving, it decreases in the same fashion for the two types of individuals with the sheltered population and the resulting interattractions between individuals. When naive and conditioned individuals are mixed, the selection of PS is not simply the average of individual preferences but rather, there is a more important weight of the naive individuals in the selection (Figure 2C). In other words, an uneven interplay between individual information and public information takes place and naive individuals act as leaders or influencers a more suitable term here, as there are no coordinated movements in this species.

In mixed groups, the minority of naive individuals starts the process of settlement, and their intrinsic preference for the PS increases the frequency of selection of this shelter. Nevertheless, the conditioned individuals influence the dynamics of the mixed groups by being slower and by avoiding the PS (owing to their past experience) (Figure 1A). The model highlights not only the critical role of naive individuals for the selection of the shelter but also the role of conditioned individuals on the rate to reach consensus. Using the estimated parameters (Table 3), the model shows that to have the CS more frequently selected, the proportion of conditioned animals should be greater than 0.8 (Figure 3). Similar results were generated with mixed groups of cockroaches-robots having the choice to settle in a dark or light shelter (Halloy et al., 2007). These robots interact with cockroaches and mimic their behavior, but with a difference: the robots are programmed to prefer the lighter shelter and the innate behavior of cockroaches being a preference for the darker shelter. By means of social interactions, robots and cockroaches of a mixed group settle together under the same shelter and, more frequently, under the lighter shelter than a group of only cockroaches. Our results show that positive feedbacks are at the origin of a diversity of outcomes. At low population sizes, the fission of the group is more frequent. Here, the positive feedbacks inside each shelter associated to the strength of interindividual attraction (*ς*) and the number of sheltered individuals is not large enough to retain them and to lead to a consensus. Reaching a consensus at large population sizes is also less frequent but for a different reason. In this case, the probability to find numerous conspecifics inside both shelters is large (Figures 4B–4D and S7–S9) leading to a decrease of the leaving probability from both shelters and favoring the fission of the group.

As seen in multiple studies, a leader can either be an individual with a low threshold (e.g., hungrier individual) or an experienced one with accurate information (on territory, migration, food patches) (Rands et al., 2003) (King and Cowlishaw, 2009; Smith et al., 2016; Sueur and Petit, 2010). The collective responses of such groups have been extensively studied in vertebrates (Couzin et al., 2005; King, 2010; Lusseau and Conradt, 2009; Pettit et al., 2015; Reebs, 2000; Roy and Bhat, 2017; Seppänen et al., 2011) but also in insects and in other invertebrates (Colasurdo and Despland, 2005; Collignon et al., 2012; Dussutour et al., 2008; Hodgkin et al., 2017; McClure et al., 2011; Plenzich and Despland, 2018; Stroeymeyt et al., 2011; Zirbes et al., 2010). However, in these cases, individual movements (including change of direction) are collectively synchronized and individuals are constantly influencing each other (Dyer et al., 2009; Lusseau and Conradt, 2009; Schultz et al., 2008), the extreme case being the queue following behavior (Fernandez and Deneubourg, 2011). In our case, sociality is linked to a particular activity and no coordinated movements are observed, the exploration of the environment being nonsocial.

From an adaptive point of view, the fact that individuals in mixed groups may choose an option which does not fit their preference or their needs, strongly suggests that benefits associated with settling with many other individuals offset the costs of a nonoptimal choice (Czaczkes, 2014; Kausrud et al., 2011), a largely discussed subject (Costa, 2006; Krause and Ruxton, 2002; Sumpter, 2010). Our theoretical prediction of the dispersion of individuals for large populations is in agreement with the benefits of aggregation or group living associated with different cooperative or Allee effects (e.g., reduction of stress) (Angulo et al., 2018; Krause and Ruxton, 2002; Yoder and Grojean, 1997). Indeed, such large populations make it possible to have numerous individuals at each site and therefore to have effective cooperation within each shelter even if the population is dispersed.

Here, we tackled an important phenomenon in social organisms: groups dynamics and decision-making when individuals possess unshared preferences. We highlighted how group dynamics are linked to the interindividual variability (Jolles et al., 2017). Finally, although more common in vertebrates, we show that influencers (or leaders) “emerge” owing to behavioral variability, mainly through a more marked behavior. Being the result of particular personality traits (Flack et al., 2012; Planas-Sitjà et al., 2015), life-history (learning, memory) (Pasquaretta et al., 2016), or temporary (physiological necessities) (Fischhoff et al., 2007; McClure et al., 2011), these behaviors are not mutually exclusive. In this context, somewhat similar results were reported for egg-laying site choice in mixed groups of trained (to an odor) and untrained fruit flies. However, these groups appear to show no consensus, the proportion of eggs laid on the “right” medium by untrained (trained) flies being positively (negatively) influenced by the number of interactions with trained (untrained) flies (Battesti et al., 2015; see also Philippe et al., 2016). These results are similar to those reported in heterospecific groups (Boulay et al., 2019; Leoncini and Rivault, 2005; Nicolis et al., 2016). For example the Diptera larvae that feed on vertebrate carrion form aggregates located at species-specific temperatures. However, several species are able to form mixed-species aggregates at temperatures which are not the thermopreferandum of each of the species (Fouche et al., 2018; Komo et al., 2021).

The results described in this article and the examples provided previously clearly suggest that the collective behaviors generated by the conflict between individual preferences and cooperative feedbacks are a widespread phenomenon. In this context, we proposed a generic script that can be at work whatever the origin of diversity be it within the same species ([epi]genetics, learning, …) or in heterospecific situations.

### Limitation of the study

All animals came from ten-year-old rearing cages, thus, consanguinity could play an important role in the cohesiveness observed between individuals. For this reason, it could be interesting to use cockroaches from other strains in the experiments. Furthermore, in this study, the controlled individual variability is induced adversely (electroshocks), and it is therefore not known if the conditioned cockroaches are less stress outside thus, their displacement is slower or if they are physiologically affected by the electricity. Among the important issues, that are not addressed here, are the ones related to the roles of starvation on social strength and of the loss of the conditioned memory during the experiments. Indeed, although there is no indication that a memory loss occurs in the short term (Balderrama, 1980; Sakura and Mizunami, 2001; Watanabe et al., 2003), in the long term, we do not exclude a gradual reversal of the conditioning, could happen (Watanabe et al., 2003). Experiments lasting several weeks would make it possible to decide whether the collective responses showed here are indeed stabilized in the long term and that a potential loss of memory would play a minor role.

## STAR★Methods

### Key resources table

**Table:** 

REAGENT or RESOURCE	SOURCE	IDENTIFIER
**Deposited data**		

Repository data from this study	https://doi.org/10.6084/m9.figshare.13221047.v1	NA

**Experimental models: Organisms/Strains**		

Periplaneta Americana	Reared at the Université Libre de Bruxelles	NA

**Software and algorithms**		

R Studio (R Core Team, 2018; R Foundation for statistical computing)	https://www.r-project.org/	NA
Fortran90	https://www.fortran90.org/	NA
Custom R and Fortran90 scripts	https://doi.org/10.6084/m9.figshare.13221047.v1	NA

### Resource availability

#### Lead contact

Information and requests for resources should be directed to and will be fulfilled by the lead contact, Mariano Calvo Martín (mcalvoma@ulb.ac.be).

#### Materials availability

No new materials were generated in this study.

#### Data and code availability

Data have been deposited at figshare and is publicly available as of the date of publication. DOIs are listed in the key resources table.

All original code has been deposited at figshare and is publicly available as of the date of publication. DOIs are listed in the key resources table.

### Experimental model and subject details

Cockroaches of the species *Periplaneta americana* were reared in an air-conditioned room at 25° C under a 12 h:12 h light:dark cycle, in breeding boxes of circa 1000 individuals in all stages of development and with both sexes mixed. The cockroaches have access to water and dog food pellets (*Tom & Co*) ad libitum. Groups of 10 nymphs (L6-L7 instar of both sexes) were kept in a box (15.5 × 11 × 6 cm) with water and food for 24 h before being conditioned or tested. Cockroaches did not have access to water or food during the experiments. The sex of L6-L7 instar of this species is not recognizable at naked eye, and therefore, the ratio male/female is unknown. However, there is no behavioral differences between the sexes at these life stage cycle.

### Method details

#### Conditioning procedure

Individuals are conditioned in groups of 10. The conditioning device (Figure S10 of the supplemental information) consists of two parts: one illuminated, 8 × 23.5 cm; 5000 ± 500 lux (4 fluorescent tube of 18 W, light comes from below through a semitransparent white plexiglass), and the other a shelter, an inverted Petri dish (diameter: 8.5 cm; 165 lux and a light spectrum pic at 650 nm) covered by a red filter film (*Rosco E+ # 019 - fire*). This dark shelter contains a cup filled with peanut butter (*Boni*) covered with a perforated plastic layer so that the individuals can only scent the peanut butter. The bottom of the shelter is an electric plate (silicate plate with copper strips) linked to a power source. A group of individuals is placed in the illuminated part and when an individual enters the shelter, an electric shock (7.5 V and 2 A) is manually delivered after 2 s. This delay allows the animal to associate the odor and the shelter. The shocked cockroach always runs out of the shelter. The procedure is applied for at least 30 min and is then continued until no individual enters the shelter for 5 consecutive min. At the end of the procedure, cockroaches were aggregated outside the shelter, this suggest that sociality is not affected by the conditioning. Thus, they are doubly conditioned: to lose their inherent preference for the odor and to spend more time outside. For example, for the conditioned condition, the group received a mean ± sd of electric shocks of 55.26 ± 14.4, with a mean ± sd duration of a conditioning trial of 2469 ± 376 s. Finally, the number of shocks administrated decreased over time (Figure S11 of the supplemental information). The preference test of a group takes place 1 to 2 h after the conditioning procedure.

#### Experimental procedure

For the experiments, we used 69 groups of 10 cockroach nymphs without any external damage. The groups are divided into 3 conditions: 15 groups of individuals without training, the naive condition (naive); 19 groups of conditioned individuals, the conditioned condition (conditioned); and 35 groups composed of 4 naive individuals and 6 conditioned individuals (mixed). For the mixed condition, conditioned individuals were marked on their pronotum with a black color dot and naive individuals with red color dot, after they were taken from their breeding box. Each group is tested in only one trial, which consists of a binary choice set-up (Figure S12 from the supplemental information). This includes two circular shelters, that are an inverted Petri dish (diameter: 8.5 cm 165 lux and light spectrum peak at 650 nm) covered by a red filter film (*Rosco E+∖# 019 - fire*) and are disposed symmetrically in a rectangular arena (35 × 23.5 × 13.4 cm; LxWxH), whose bottom is covered with a white paper layer covering the floor as well inside the shelters, this is changed after every trial. A light source (8 fluorescent tubes lamps – 80 W/T5 SYLVANIA), placed 190 cm above the arena, provides a homogeneous illumination intensity at the ground level (500 ± 50 lux, with a peak at 577 nm). The shelters are as described in the conditioning procedure section. Under each shelter, there is a cup (diameter: 0.16 cm) covered with a plastic film perforated with a needle, to prevent cockroaches to have direct contact with the interior of the cups. One of the shelters' cups is filled with peanut butter (*Boni*), this being the odorous shelter (PS). For the other, the control shelter (CS), the cup is empty. The group is released into the center of the arena all at once and left to explore and seek shelter for 24 hr.

#### Simulation

Simulations are performed using R studio (R Core Team, 2018, R Foundation for Statistical Computing, https://www.r-project.org/) and Fortran90 (https://www.fortran90.org/).

### Quantification and statistical analysis

Trials are recorded by a video camera (*Logitech C920*), located over the set-up, at 17 frames per s for the first 3 h of the trial. The red filter covering the shelter, allow the video camera to detect the cockroaches inside the shelters. The distribution of individuals among the shelters is recorded every time-step (10 min) during the first 3 h and 24 h after the beginning of the trial by counting the number of individuals in each shelter.

Data and statistical analysis are performed using R studio (R Core Team, 2018, R Foundation for Statistical Computing, https://www.r-project.org/). The significance of the statistical tests is fixed to α = 0.05. As the dynamics of sheltering process of gregarious and social insects are usually nonlinear and/or their variances vary over time, we used permutation and resampling tests (Good, 2005, 2006). Comparisons of the sheltering process (total sheltered population) between conditions, at every time-step, are made with resampling tests: 10,000 iterations of resampling the number of replicates of a condition with the data of another one. The null hypothesis is that the total sheltered population, at a particular time, does not depend on the condition. Thus, the observed total sheltered population of a condition is within 95% of the iterated distribution from another. Otherwise, the null hypothesis is rejected, and the alternative is that the sheltering processes are different between conditions. To test whether the selection of a shelter is socially motivated and does not result only on individual preferences, we compared the observed sheltered distribution in the PS (for the naive and the mixed) and in the CS (for the conditioned) for each time-step, with a binomial distribution with a constant probability to select the shelter given by the observed fraction of individuals under a particular shelter at the given time-step (Farr, 1978). This probability is the average fraction, calculated over all the experiences of a condition, of individuals housed in the PS or the CS (for example: $\frac{Total\;number\;of\;individuals\;in\;the\;PS}{Total\;number\;of\;sheltered\;individuals}$). To define the expected distribution under nonsocial behavior, we compute the theoretical variance of the sheltered population in the PS or CS (depending on the condition) expected for individuals without interactions. We then perform 10,000 iterations, considering that the individual mean probability of sheltering was equal to the proportion of the population in the PS or the CS. The results of the iterations are weighted based on the variance of the number of individuals in the shelters (PS or CS). The null hypothesis is that the selection of a shelter is nonsocial and follows a binomial distribution, thus the observed variance should be within 95% of the distribution of the simulation. Otherwise, the null hypothesis is rejected, and the alternative is that shelter selection is mediated by the presence of conspecifics, and thus, selection of a shelter is made by the group and not individually. If this first test highlights or not sociability, it does not show a consensus. These consensuses lead to bimodal distributions (in binary choices), as two responses are possible. Our definition of consensus implies that all or the majority of individuals exhibit the same choice, and the formation of only one aggregate. Bimodal distributions and the presence of peaks are tested and obtained at each time-step using the Cramer-von Mises test with the Fisher and Marron method from the R package “Multimode” (Ameijeiras-Alonso et al., 2021).

We consider that the shelter containing the largest number of individuals at a specific time step is the winning one for that specific time. Tw is defined as the time it takes for one of the shelters to become the most populated one until the end of the trial (24 h). A survival analysis (log rank test) is performed on this variable. Indeed, the inverse of Tw can be viewed as the mean probability per unit time that a shelter becomes the most populated one until the end. Moreover, we use the Spearman correlation tests to quantify the correlation between the population of a winning shelter of times 180 and 1440 min and for the mixed condition, the correlation between the numbers of naive and conditioned individuals present in the same shelter. We use permutation tests (10,000 iterations) to compare the sheltered population between the PS and CS within each condition and at each time step (Good, 2005): at each iteration, to recalculate the mean sheltered population in the PS (for the naive and mixed conditions) and the CS (for the conditioned condition), we randomly permute the observed sheltered population between of the PS and the CS for each trial and at every time step of the 3 conditions. The null hypothesis corresponding to the lack of preferences between the shelters is rejected when the observed mean proportion of the PS(CS) is out of the 95% of the permutation distribution of the mean sheltered population of the PS(CS). Thus, the alternative hypothesis is that, depending on the condition and the time, there is an uneven preference between the shelters. Finally, the influence of the proportion of conditioned individuals in a group regarding the selection of the PS is analyzed by nonlinear least square model using the Levenberg-Marquardt algorithm (Moré, 1978).

Our model assumes that the total number of entering individuals in both shelters (N_ps_ + N_cs_) per unit of time is proportional to θ and to the number of individuals outside the shelters. The inferior limit of θ can be approximated if we neglect the exits from the shelters (ρ = 0). Thus, the mean total sheltered population as a function of time based on Equation 2, of the section Mechanism of shelter selection and model becomes:

(Equation 4) $$N\left(1-e^{-\theta t}\right)$$

where N is the total population (here N = 10) and t is the time (seconds). This equation (Equation 4) is fitted with a nonlinear least square model using the Levenberg-Marquardt fitting algorithm (Moré, 1978) and allows us to estimate the minimal values of θ for the naive and conditioned conditions (see Table 2).

The system of differential equations Equation 5 based on Equations 2 and 3 of the main text is the analytical equivalent of the simulations from section Mechanism of shelter selection and model. The state of the system is described in terms of a probability function *P(i, j)* which at time t, i is the number of individuals sheltered in PS and j in CS (*0 ≤ i ≥ N, 0 ≤ j ≥ N, 0 ≤ i + j ≥ N)*. Each differential equation $\frac{dP\left(i,j\right)}{dt}$ describes the time evolution of the probability *P(i, j)* that the system occupies the state *(i, j)*. The equation counts the transitions leading the system to the state*(i, j)* and those removing it from this state. In our case, the transitions depend on both the probabilities of joining the shelters PS or CS ($\theta \mu_{ps}$$\theta \mu_{cs}$) and the probability of leaving it ($\rho e^{-\varsigma \left(i-1\right)}$, $\rho e^{-\varsigma \left(j-1\right)}$).

(Equation 5) $$\begin{array}{l} \frac{dP\left(i,j\right)}{dt}=-\left(\theta \mu_{ps}\left(N-i-j\right)+\theta \mu_{cs}\left(N-i-j\right)+\rho ie^{-\varsigma \left(i-1\right)}+\rho je^{-\varsigma \left(j-1\right)}\right)P\left(i,j\right)+\theta \mu_{ps}\left(N-\left(i-1\right)-j\right)P\left(i-1,j\right)+\theta \mu_{cs}\left(N-i-\left(j-1\right)\right)P\left(i,j-1\right)+\rho ie^{-\varsigma \left(i\right)}P\left(i+1,j\right)+\rho je^{-\varsigma \left(j\right)}P\left(i,j+1\right)\\ \mu_{ps}+\mu_{cs}=1 \end{array}$$

This system of equations is particularly effective for quickly obtaining the theoretical distribution of the different states. Indeed, by numerically integrating the master equation Equation 5 for 24 h and comparing the results of numerous combinations of parameter values with the experimental one, we are able to identify the model parameters that are compatible with the experimental naive and conditioned conditions. The criteria for comparison of the experimental and of the theoretical data are 1) at 24 h the 99% of the total population is sheltered; 2) 100% of the total sheltered population is in the same shelter (bimodal distribution), and 3) the experimental proportion of the selected shelter is respected (naive individuals PS 0.8, CS 0.2; conditioned individuals PS 0.4, CS 0.6). We can also obtain the exact steady state solution of Equation 5 (time = ∞):

(Equation 6) $$\begin{array}{l} P\left(i,j\right)\;=P\left(0,0\right)\frac{N!}{i!j!\left(N-i-j\right)!}{\left(\frac{\theta }{\rho }\right)}^{i+j}\mu_{cs}^{j}\mu_{ps}^{i}e^{\frac{\varsigma \left(j-1\right)j+\varsigma \left(i-1\right)i}{2}}=P\left(0,0\right)A_{ij}\\ P\left(0,0\right)=\frac{1}{\sum_{i=0}^{N}\sum_{j=0}^{N}A_{ij}}\;0\leq i\leq N,0\leq j\leq N,0\leq i+j\leq N \end{array}$$

In the case of ɩ = 0, Equation 6 corresponds to a multinomial distribution and therefore to a distribution of nonsocial individuals:

Probability to be out of the shelters: $\frac{\theta }{\theta \;+\;\rho }$

Probability to be within the CS: $\frac{\theta \mu_{cs}}{\theta +\rho }$

Probability to be within the PS: $\frac{\theta \mu_{ps}}{\theta +\rho }$

Further approximation of the parameter values, from Table 2, is made through stochastic simulations (10,000 realizations) of the model and subsequent resampling test (10,000 iterations) to compare the outputs of the simulations (simulated results) to the experimental results for each time-step. This test, based on (Good, 2006), consists in randomly generate sets of simulated results (simulated replicates: corresponding to the number of replicates for each experimental condition) and comparing them to the experimental results, at every time step. At each iteration and for each time step, the sheltered proportion in the PS (for the naive and the mixed simulated conditions) and in the CS (for the conditioned condition) is calculated. The null hypothesis is that the experimental sheltered proportion in the PS(CS) is within the 95% of the sets of the simulated sheltered proportion in the PS(CS). Otherwise, the null hypothesis is rejected and the simulated data do not correspond to the experimental ones. The combinations of the parameters values that yield the best results of the test (Figure S6A) are those of Table 3.

## Footnotes

Supplemental information can be found online at https://doi.org/10.1016/j.isci.2021.102723.

## References

[bib1] Ame J.-M., Halloy J., Rivault C., Detrain C., Deneubourg J.L. (2006). Collegial decision making based on social amplification leads to optimal group formation. Proc. Natl. Acad. Sci. U S A.

[bib2] Ameijeiras-Alonso J., Crujeiras R.M., Rodríguez-Casal A. (2021). Multimode: an R package for mode assessment. J. Stat. Softw..

[bib3] Angulo E., Luque G.M., Gregory S.D., Wenzel J.W., Bessa-Gomes C., Berec L., Courchamp F. (2018). Review: Allee effects in social species. J. Anim. Ecol..

[bib4] Balderrama N. (1980). One trial learning in the American cockroach, Periplaneta americana. J. Insect Physiol..

[bib5] Battesti M., Pasquaretta C., Moreno C., Teseo S., Joly D., Klensch E., Petit O., Sueur C., Mery F. (2015). Ecology of information: social transmission dynamics within groups of non-social insects. Proc. R. Soc. B Biol. Sci..

[bib6] Bell W.J., Adiyodi K.G. (1981). The American Cockroach.

[bib7] Bell W.J., Roth L.M., Nalepa C.A. (2007). Cockroaches: Ecology, Behavior, and Natural History.

[bib8] Boulay J., Aubernon C., Ruxton G.D., Hédouin V., Deneubourg J.-L., Charabidzé D. (2019). Mixed-species aggregations in arthropods. Insect Sci..

[bib9] Bourjade M., Thierry B., Maumy M., Petit O. (2009). Decision-making in przewalski horses (equus ferus przewalskii) is driven by the ecological contexts of collective movements. Ethology.

[bib10] Calvo Martín M., Nicolis S.C., Planas-Sitjà I., Deneubourg J.L. (2019). Conflictual influence of humidity during shelter selection of the American cockroach (Periplaneta americana). Sci. Rep..

[bib11] Camazine S., Deneubourg J.-L., Franks N.R., Sneyd J., Bonabeau E., Theraulaz G. (2003). Self-organization in Biological Systems.

[bib12] Cardé R.T., Willis M.A. (2008). Navigational strategies used by insects to find distant, wind-borne sources of odor. J. Chem. Ecol..

[bib13] Carter K.D., Seddon J.M., Frère C.H., Carter J.K., Goldizen A.W. (2013). Fission-fusion dynamics in wild giraffes may be driven by kinship, spatial overlap and individual social preferences. Anim. Behav..

[bib14] Cloudsley-Thompson J.L., Constantinou C. (1987). Humidity reactions and aggregation in woodlice (isopoda, oniscoidea). Crustaceana.

[bib15] Colasurdo N., Despland E. (2005). Social cues and following behavior in the forest tent caterpillar. J. Insect Behav..

[bib16] Collignon B., Deneubourg J.L., Detrain C. (2012). Leader-based and self-organized communication: modelling group-mass recruitment in ants. J. Theor. Biol..

[bib17] Conradt L., Roper T.J. (2005). Consensus decision making in animals. Trends Ecol. Evol..

[bib18] Conradt L., Roper T.J. (2007). Democracy in animals: the evolution of shared group decisions. Proc. R. Soc. B Biol. Sci..

[bib19] Copp N.H. (1983). Temperature-dependent behaviours and cluster formation by aggregating ladybird beetles. Anim. Behav..

[bib20] Costa J.T. (2006). The Other Insect Societies.

[bib21] Couzin I.D., Krause J., Franks N.R., Levin S.A. (2005). Effective leadership and decision-making in animal groups on the move. Nature.

[bib22] Croney C.C., Newberry R.C. (2007). Group size and cognitive processes. Appl. Anim. Behav. Sci..

[bib23] Czaczkes T.J. (2014). How to not get stuck-Negative feedback due to crowding maintains flexibility in ant foraging. J. Theor. Biol..

[bib24] Duffy J.E., Thiel M. (2007). Evolutionary Ecology of Social and Sexual Systems: Crustaceans as Model Organisms.

[bib25] Dussutour A., Nicolis S.C., Despland E., Simpson S.J. (2008). Individual differences influence collective behaviour in social caterpillars. Anim. Behav..

[bib26] Dyer J.R.G., Johansson A., Helbing D., Couzin I.D., Krause J. (2009). Leadership, consensus decision making and collective behaviour in humans. Philos. Trans. R. Soc. B Biol. Sci..

[bib27] Eckholm B.J., Anderson K.E., Weiss M., DeGrandi-Hoffman G. (2011). Intracolonial genetic diversity in honeybee (Apis mellifera) colonies increases pollen foraging efficiency. Behav. Ecol. Sociobiol..

[bib28] Endres T., Fendt M. (2007). Conditioned behavioral responses to a context paired with the predator odor trimethylthiazoline. Behav. Neurosci..

[bib29] Farr J.A. (1978). Orientation and social behavior in the supralittoral isopod Ligia exotica (Crustacea: oniscoidea). Bull. Mar. Sci..

[bib30] Fernandez A.A., Deneubourg J.L. (2011). On following behaviour as a mechanism for collective movement. J. Theor. Biol..

[bib31] Feussner I., Wasternack C. (2002). The lipoxygenase pathway. Annu. Rev. Plant Biol..

[bib32] Fischhoff I.R., Sundaresan S.R., Cordingley J., Larkin H.M., Sellier M.-J., Rubenstein D.I. (2007). Social relationships and reproductive state influence leadership roles in movements of plains zebra. Equus Burchellii. Anim. Behav..

[bib33] Fitzgerald T.D., Pescador-Rubio A. (2002). The role of tactile and chemical stimuli in the formation and maintenance of the processions of the social caterpillar Hylesia lineata (Lepidoptera: saturniidae). J. Insect Behav..

[bib34] Flack A., Pettit B., Freeman R., Guilford T., Biro D. (2012). What are leaders made of? The role of individual experience in determining leader–follower relations in homing pigeons. Anim. Behav..

[bib35] Fouche Q., Hedouin V., Charabidze D. (2018). Communication in necrophagous Diptera larvae: interspecific effect of cues left behind by maggots and implications in their aggregation. Sci. Rep..

[bib36] Full R.J., Tu M.S. (1991). Mechanics of a rapid running insect: two-, four- and six-legged locomotion. J. Exp. Biol..

[bib37] Good P.I. (2005). Permutation, Parametric, and Bootstrap Tests of Hypotheses.

[bib38] Good P.I. (2006). Resampling Methods: A Practical Guide to Data Analysis.

[bib39] Günzel Y., McCollum J., Paoli M., Galizia C.G., Petelski I., Couzin-Fuchs E. (2021). Social modulation of individual preferences in cockroaches. iScience.

[bib40] Halloy J., Sempo G., Caprari G., Rivault C., Asadpour M., Tache F., Said I., Durier V., Canonge S., Ame J.M. (2007). Social integration of robots into groups of cockroaches to control self-organized choices. Science.

[bib41] Haney J., Lukowiak K. (2001). Context learning and the effect of context on memory retrieval in Lymnaea. Learn. Mem..

[bib42] Hodgkin L.K., Symonds M.R.E., Elgar M.A. (2017). Leadership through knowledge and experience in a social sawfly. Anim. Behav..

[bib43] Jeanson R., Deneubourg J.-L., Grimal A., Theraulaz G. (2004). Modulation of individual behavior and collective decision-making during aggregation site selection by the ant Messor barbarus. Behav. Ecol. Sociobiol..

[bib44] Jeanson R., Clark R.M., Holbrook C.T., Bertram S.M., Fewell J.H., Kukuk P.F. (2008). Division of labour and socially induced changes in response thresholds in associations of solitary halictine bees. Anim. Behav..

[bib45] Jolles J.W., Boogert N.J., Sridhar V.H., Couzin I.D., Manica A. (2017). Consistent individual differences drive collective behavior and group functioning of schooling fish. Curr. Biol..

[bib46] Karimifar N., Gries R., Khaskin G., Gries G. (2011). General food semiochemicals attract omnivorous German cockroaches, blattella germanica. J. Agric. Food Chem..

[bib47] Kausrud K.L., Grégoire J.-C., Skarpaas O., Erbilgin N., Gilbert M., Økland B., Stenseth N.C. (2011). Trees wanted—dead or alive! Host selection and population dynamics in tree-killing bark beetles. PLoS One.

[bib48] Kerth G., König B. (1999). Fission, fusion and nonrandom associations in female Bechstein’s bats (Myotis bechsteinii). Behaviour.

[bib49] King A.J. (2010). Follow me! I’m a leader if you do; I’m a failed initiator if you don’t?. Behav. Process..

[bib50] King A.J., Cowlishaw G. (2009). Leaders, followers, and group decision-making. Commun. Integr. Biol..

[bib51] Koehl M.A.R. (2006). The fluid mechanics of arthropod sniffing in turbulent odor plumes. Chem. Senses.

[bib52] Komo L., Hedouin V., Charabidze D. (2021). Benefits of heterospecific aggregation on necromass: influence of temperature, group density, and composition on fitness-related traits. Insect Sci..

[bib53] Krafft B., Horel A., Julita J.-M. (1986). Influence of food supply on the duration of the gregarious phase of a maternal-social spider, coelotes terrestris (araneae, agelenidae). J. Arachnol..

[bib54] Krause J., Ruxton G.D. (2002). Living in Groups.

[bib55] Krause J., Godin J.-G.J., Brown D. (1996). Phenotypic variability within and between fish shoals. Ecology.

[bib56] Laland K.N. (2004). Social learning strategies. Learn. Behav..

[bib57] Laurent Salazar M.-O., Nicolis S.C., Calvo Martín M., Sempo G., Deneubourg J.-L., Planas-Sitjà I. (2017). Group choices seemingly at odds with individual preferences. R. Soc. Open Sci..

[bib58] Lazzari C.R., Lorenzo M.G. (2009). Exploiting triatomine behaviour: alternative perspectives for their control. Mem. Inst. Oswaldo Cruz.

[bib59] Leoncini I., Rivault C. (2005). Could species segregation be a consequence of aggregation processes? Example of Periplaneta americana (L.) and P. fuliginosa (serville). Ethology.

[bib60] Lihoreau M., Costa J.T., Rivault C. (2012). The social biology of domiciliary cockroaches: colony structure, kin recognition and collective decisions. Insectes Soc..

[bib61] Lusseau D., Conradt L. (2009). The emergence of unshared consensus decisions in bottlenose dolphins. Behav. Ecol. Sociobiol..

[bib62] Mathis A., Chivers D.P., Smith R.J.F. (1996). Cultural transmission of predator recognition in fishes: intraspecific and interspecific learning. Anim. Behav..

[bib63] McClure M., Ralph M., Despland E. (2011). Group leadership depends on energetic state in a nomadic collective foraging caterpillar. Behav. Ecol. Sociobiol..

[bib64] Moré J.J. (1978). The Levenberg-Marquardt Algorithm: Implementation and Theory.

[bib65] Nalyanya G., Schal G. (2001). Evaluation of attractants for monitoring populations of the German cockroach (Dictyoptera: blattellidae). J. Econ. Entomol..

[bib66] Nicolis G., Prigogine I. (1977). Self-Organization in Non-equilibrium Systems : From Dissipative Structures to Order through Fluctuations.

[bib67] Nicolis S.C., Halloy J., Deneubourg J.L. (2016). Transition between segregation and aggregation: the role of environmental constraints. Sci. Rep..

[bib68] Nicolis S.C., Pin A., Calvo Martín M., Planas-Sitjà I., Deneubourg J.-L. (2020). Sexual group composition and shelter geometry affect collective decision-making: the case of Periplaneta americana. Insectes Soc..

[bib69] Paoli M., Nishino H., Couzin-Fuchs E., Galizia C.G. (2020). Coding of odour and space in the hemimetabolous insect Periplaneta americana. J. Exp. Biol..

[bib70] Pasquaretta C., Battesti M., Klenschi E., Bousquet C.A.H., Sueur C., Mery F. (2016). How social network structure affects decision-making in Drosophila melanogaster. Proc. R. Soc. B Biol. Sci..

[bib71] Petit O., Bon R. (2010). Decision-making processes: the case of collective movements. Behav. Process..

[bib72] Pettit B., Ákos Z., Vicsek T., Biro D. (2015). Speed determines leadership and leadership determines learning during pigeon flocking. Curr. Biol..

[bib73] Philippe A.-S., Jeanson R., Pasquaretta C., Rebaudo F., Sueur C., Mery F. (2016). Genetic variation in aggregation behaviour and interacting phenotypes in Drosophila. Proc. R. Soc. B Biol. Sci..

[bib74] Planas-Sitjà I., Deneubourg J.-L., Gibon C., Sempo G. (2015). Group personality during collective decision-making: a multi-level approach. Proc. R. Soc. B Biol. Sci..

[bib75] Plenzich C., Despland E. (2018). Host-plant mediated effects on group cohesion and mobility in a nomadic gregarious caterpillar. Behav. Ecol. Sociobiol..

[bib76] Prokopy R.J., Roitberg B.D. (2001). Joining and avoidance behavior in nonsocial insects. Annu. Rev. Entomol..

[bib77] Rands S.A., Cowlishaw G., Pettifor R.A., Rowcliffe J.M., Johnstone R.A. (2003). Spontaneous emergence of leaders and followers in foraging pairs. Nature.

[bib78] Ravary F., Lecoutey E., Kaminski G., Châline N., Jaisson P. (2007). Individual experience alone can generate lasting division of labor in ants. Curr. Biol..

[bib79] Reebs S.G. (2000). Can a minority of informed leaders determine the foraging movements of a fish shoal?. Anim. Behav..

[bib80] Robinson G.E. (1985). Effects of a juvenile hormone analogue on honey bee foraging behaviour and alarm pheromone production. J. Insect Physiol..

[bib81] Rosenthal S.B., Twomey C.R., Hartnett A.T., Wu H.S., Couzin I.D. (2015). Revealing the hidden networks of interaction in mobile animal groups allows prediction of complex behavioral contagion. Proc. Natl. Acad. Sci. U S A.

[bib82] Roy T., Bhat A. (2017). Social learning in a maze? Contrasting individual performance among wild zebrafish when associated with trained and naïve conspecifics. Behav. Process..

[bib83] Sakura M., Mizunami M. (2001). Olfactory learning and memory in the cockroach Periplaneta americana. Zool. Sci..

[bib84] Scheid C., Noë R. (2010). The performance of rooks in a cooperative task depends on their temperament. Anim. Cogn..

[bib85] Schultz K.M., Passino K.M., Seeley T.D. (2008). The mechanism of flight guidance in honeybee swarms: subtle guides or streaker bees?. J. Exp. Biol..

[bib86] Seppänen J.-T.T., Forsman J.T., Mönkkönen M., Krams I., Salmi T. (2011). New behavioural trait adopted or rejected by observing heterospecific tutor fitness. Proc. R. Soc. B Biol. Sci..

[bib87] Smith J.E., Gavrilets S., Mulder M.B., Hooper P.L., El Mouden C., Nettle D., Hauert C., Hill K., Perry S., Pusey A.E. (2016). Leadership in mammalian societies: emergence, distribution, power, and payoff. Trends Ecol. Evol..

[bib88] Steinbrecht R.A., Bock G.R., Cardew G. (2007). Structure and function of insect olfactory sensilla. Ciba Foundation Symposium 200.

[bib89] Strandburg-Peshkin A., Twomey C.R., Bode N.W.F., Kao A.B., Katz Y., Ioannou C.C., Rosenthal S.B., Torney C.J., Wu H.S., Levin S.A. (2013). Visual sensory networks and effective information transfer in animal groups. Curr. Biol..

[bib90] Stroeymeyt N., Franks N.R., Giurfa M. (2011). Knowledgeable individuals lead collective decisions in ants. J. Exp. Biol..

[bib91] Strutz A., Soelter J., Baschwitz A., Farhan A., Grabe V., Rybak J., Knaden M., Schmuker M., Hansson B.S., Sachse S. (2014). Decoding odor quality and intensity in the Drosophila brain. Elife.

[bib92] Sueur C., Petit O. (2010). Signals use by leaders in Macaca tonkeana and Macaca mulatta: group-mate recruitment and behaviour monitoring. Anim. Cogn..

[bib93] Sumpter D.J. (2006). The principles of collective animal behaviour. Philos. Trans. R. Soc. B Biol. Sci..

[bib94] Sumpter D.J.T. (2010). Collective Animal Behavior.

[bib95] Verhoef H.A., Witteveen J. (1980). Water balance in Collembola and its relation to habitat selection; cuticular water loss and water uptake. J. Insect Physiol..

[bib96] Ward A., Webster M. (2016). Collective decision-making. Sociality: The Behaviour of Group-Living Animals.

[bib97] Watanabe H., Kobayashi Y., Sakura M., Matsumoto Y., Mizunami M. (2003). Classical olfactory conditioning in the cockroach Periplaneta americana. Zool. Sci..

[bib98] Webster M.M., Ward A.J.W. (2011). Personality and social context. Biol. Rev..

[bib99] Weidenmuller A. (2004). The control of nest climate in bumblebee (Bombus terrestris) colonies: interindividual variability and self reinforcement in fanning response. Behav. Ecol..

[bib100] Yoder J.A., Grojean N.C. (1997). Group influence on water conservation in the giant Madagascar hissing-cockroach, Gromphadorhina portentosa (Dictyoptera: blaberidae). Physiol. Entomol..

[bib101] Zimer-Faust R.K., Tyre J.E., Case J.F. (1985). Chemical attraction causing aggregation in the spiny lobster, panulirus interruptus (randall), and its probable ecological significance. Biol. Bull..

[bib102] Zirbes L., Deneubourg J.-L., Brostaux Y., Haubruge E. (2010). A new case of consensual decision: collective movement in earthworms. Ethology.

