# Supplementary material for: Consensus driven by a minority in heterogenous groups of the cockroach Periplaneta americana
Source: iScience. 2021 Jun 12;24(7):102723. doi: 10.1016/j.isci.2021.102723 (PMC8254023; doi:10.1016/j.isci.2021.102723)

iScience, Volume *24*

Supplemental information

Consensus driven by a minority in heterogenous groups of the cockroach *Periplaneta americana*

Mariano Calvo Martín, Max Eeckhout, Jean-Louis Deneubourg, and Stamatios C. Nicolis

Supplemental Information

Figure S1. Result of the resampling test, related to section Global dynamics.

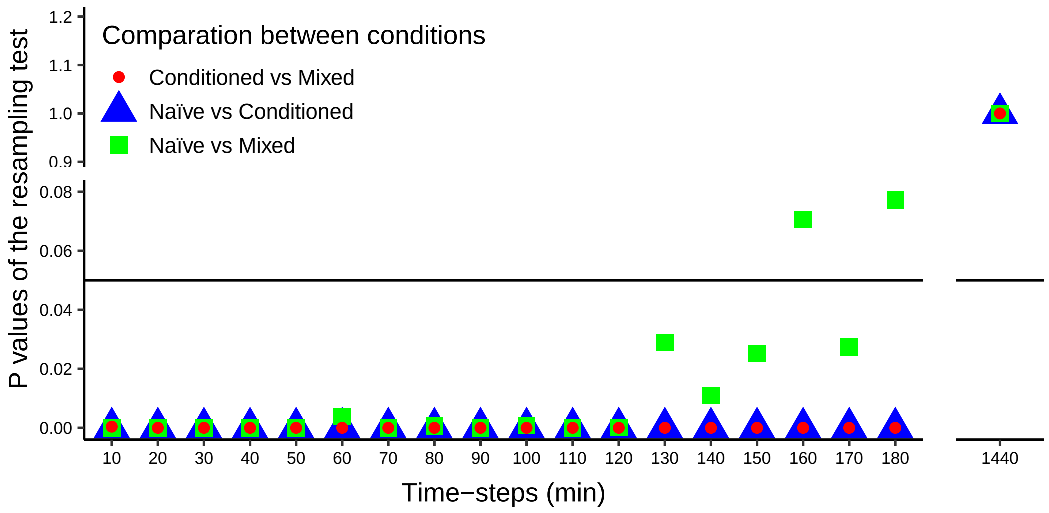

P values of the resampling test (10000 iterations) every 10 *minutes* for the first 3 *hours* and at 24 *hours*, between the conditions: naïve and conditioned (orange triangle); the naïve and mixed (red square) and the conditioned and mixed (blue circle). Solid horizontal line: significance value $\alpha = 0.05$.

Table S1. Distribution between the shelter over time, related to the figure $1B-D$ in section collective choice and consensus.

Time	Condition	Statistic F	P	Estimated modes
10	Naïve	0.109	0.558	3.458
20	Naïve	0.112	0.532	5.354
30	Naïve	0.155	0.166	5.9
40	Naïve	0.177	0.082	7.437
50	Naïve	0.187	0.056	7.898
60	Naïve	0.168	0.106	7.604
70	Naïve	0.129	0.414	8.543
80	Naïve	0.231	0.026	-5.445 ; 8.719
90	Naïve	0.264	0.016	-2.99 ; 8.768
100	Naïve	0.321	0	-7.687 ; 9.169
110	Naïve	0.343	0	-8.021 ; 9.122
120	Naïve	0.322	0	-4.77 ; 9.122
130	Naïve	0.292	0	-9.465 ; 9.245
140	Naïve	0.287	0.012	-8.493 ; 9.304
150	Naïve	0.344	0	-9.5 ; 9.389
160	Naïve	0.335	0	-9.5 ; 9.337
170	Naïve	0.357	0.004	-10 ; 9.827
180	Naïve	0.317	0	-9 ; 9.459
1440	Naïve	0.665	0	-9.693 ; 9.863
10	Conditioned	0.105	0.598	0.851
20	Conditioned	0.148	0.066	-1.512
30	Conditioned	0.160	0.058	-1.327

Continued on next page

Table 1 – continued from previous page

Time	Condition	Statistic F	P	Estimated modes
40	Conditioned	0.149	0.07	0.594
50	Conditioned	0.118	0.338	-0.233
60	Conditioned	0.110	0.502	-0.77
70	Conditioned	0.114	0.476	-5.761
80	Conditioned	0.119	0.33	-4.788
90	Conditioned	0.094	0.964	-6.73
100	Conditioned	0.107	0.668	-6.106
110	Conditioned	0.108	0.612	-6.136
120	Conditioned	0.127	0.236	-7.138
130	Conditioned	0.111	0.566	-7.636
140	Conditioned	0.153	0.072	-7.218
150	Conditioned	0.182	0.028	-9.034 ; 6.041
160	Conditioned	0.217	0.002	-8.578 ; 7.632
170	Conditioned	0.249	0	-8.571 ; 8.084
180	Conditioned	0.268	0	-8.458 ; 8.998
1440	Conditioned	0.557	0	-10 ; 10
10	Mixed	0.149	0.066	1.443
20	Mixed	0.128	0.218	0.846
30	Mixed	0.110	0.61	0.095
40	Mixed	0.111	0.534	1.823
50	Mixed	0.121	0.336	3.565
60	Mixed	0.114	0.576	5.6
70	Mixed	0.182	0.022	-5.996 ; 6.353
80	Mixed	0.146	0.15	6.393
90	Mixed	0.166	0.054	5.929
100	Mixed	0.183	0.018	-8.356 ; 7.182
110	Mixed	0.237	0.004	-8.104 ; 8.349
120	Mixed	0.219	0.006	-8.567 ; 7.236
130	Mixed	0.291	0.002	-8.439 ; 6.954
140	Mixed	0.339	0	-8.866 ; 7.818
150	Mixed	0.315	0	-8.235 ; 7.98
160	Mixed	0.332	0	-8.022 ; 7.224
170	Mixed	0.325	0	-8.468 ; 8.653
180	Mixed	0.327	0	-8.429 ; 7.962
1440	Mixed	0.750	0	-9.894 ; 9.826

Results of the Cramer-von Mises test and estimated modes of the difference between the sheltered population in the PS and in the CS.

Figure S2. Survival analysis between shelters and between conditions, related to section collective choice and consensus.

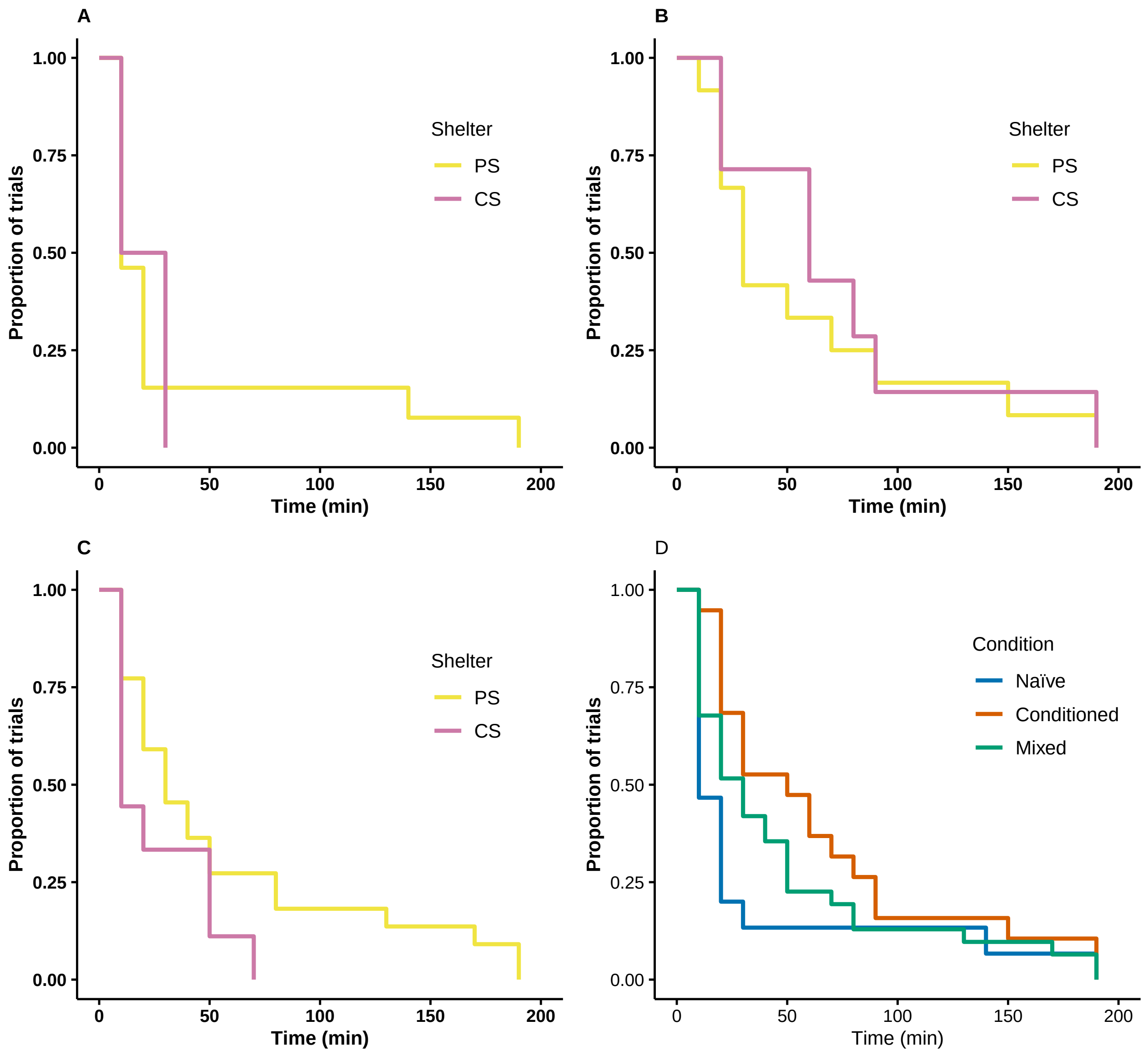

Proportion of trials as a function of the time T_w needed to become the winning shelter (survival curve). A-C) Between the PS (yellow) and CS (violet). A) Naïve condition. B) Conditioned condition. C) Mixed condition. D) Between the naïve (blue), the conditioned (orange) and the mixed (green) conditions. In relation to collective choice and consensus section.

Table S2. Linear regression of the sheltered proportion in the PS over time, related to section Shelter preference and mechanisms.

Condition	Parameter	Estimate	Std. Error	t statistic	P
Naïve	Intercept	0.78	0.02	33.4	<0.0001
	Slope	1.9 x10-5	6.7 x10-5	0.3	0.78
Conditioned	Intercept	0.39	0.03	13.34	<0.0001
	Slope	-1.8 x10-5	7.8 x10-5	-0.24	0.81
Mixed	Intercept	0.63	0.02	33.9	<0.0001
	Slope	3.8 x10-6	5.2 x10-5	0.07	0.94

Estimated parameters of the linear regression for the proportion of the population in the PS over time for the three conditions. In relation to section.

Figure S3. Sheltered proportion in the PS over time, related to section Shelter preference and mechanisms.

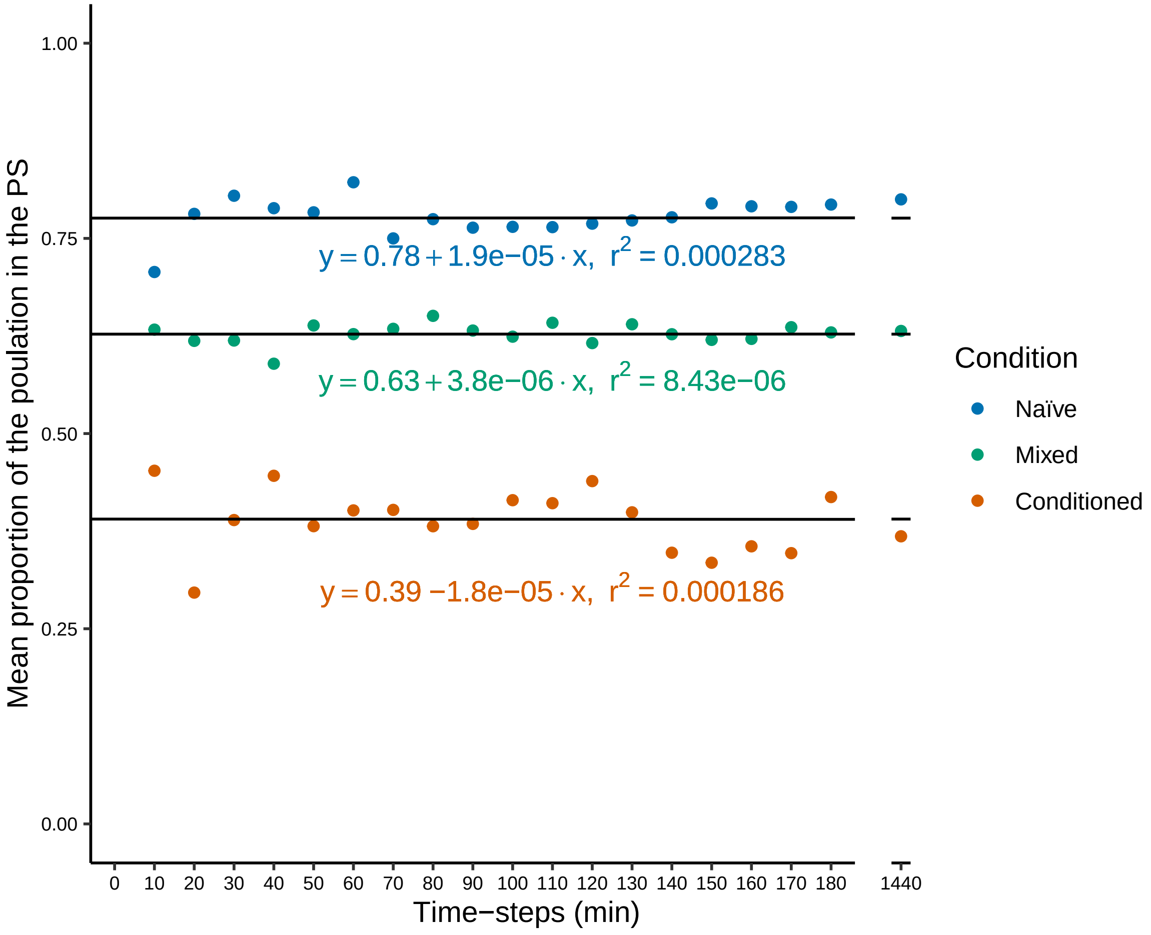

Mean proportion of the sheltered population in the PS ($\frac{N_{ps}}{N_{ps}+N_{cs}}$) over time (*minutes*) for the naïve (blue), the conditioned (orange) and the mixed conditions (green) and their respective linear fitting (table S2).

Table S3. Non-linear fitting from *eq.* 1, of the main text, related to section Shelter preference and mechanisms.

Time	Estimated	Confidence interval	Parameter	t statistic	P
10	1.619	0.604 ; 3.442	α	2.75	0.0077
20	1.707	0.658 ; 3.61	α	2.763	0.0074
30	1.006	0.064 ; 2.586	α	1.914	0.0626
40	1.615	0.581 ; 3.913	α	2.627	0.0112
50	1.636	0.605 ; 3.464	α	2.696	0.0089
60	1.731	0.653 ; 3.724	α	2.735	0.008
70	1.513	0.532 ; 3.17	α	2.609	0.0112
80	1.693	0.551 ; 3.82	α	2.46	0.0165
90	1.568	0.592 ; 3.309	α	2.683	0.0096
100	1.292	0.432 ; 2.612	α	2.563	0.0129
110	1.564	0.627 ; 3.224	α	2.874	0.0056
120	1.673	0.678 ; 3.37	α	2.79	0.007
130	1.346	0.416 ; 2.95	α	2.527	0.0141
140	1.602	0.595 ; 3.446	α	2.745	0.0078
150	1.468	0.511 ; 3.124	α	2.664	0.0097
160	1.368	0.431 ; 2.927	α	2.522	0.0141
170	1.448	0.474 ; 3.138	α	2.571	0.0124
180	1.309	0.407 ; 2.733	α	2.508	0.0146
1440	1.514	0.534 ; 3.215	α	2.663	0.0097
10	0.208	0.065 ; 0.451	β	2.573	0.0123
20	0.224	0.075 ; 0.482	β	2.619	0.0109
30	0.098	-0.062 ; 0.323	β	1.18	0.245
40	0.213	0.052 ; 0.553	β	2.296	0.0256
50	0.210	0.064 ; 0.454	β	2.521	0.0141
60	0.219	0.067 ; 0.486	β	2.526	0.014
70	0.176	0.036 ; 0.394	β	2.213	0.0304
80	0.211	0.05 ; 0.493	β	2.254	0.0275
90	0.192	0.047 ; 0.431	β	2.315	0.0243
100	0.153	0.023 ; 0.336	β	2.103	0.0396
110	0.188	0.05 ; 0.412	β	2.445	0.0175
120	0.201	0.057 ; 0.429	β	2.423	0.0183
130	0.157	0.019 ; 0.372	β	2.071	0.0426
140	0.189	0.044 ; 0.435	β	2.336	0.0227
150	0.178	0.041 ; 0.397	β	2.336	0.0225
160	0.160	0.023 ; 0.368	β	2.108	0.0389
170	0.166	0.027 ; 0.388	β	2.146	0.0356
180	0.149	0.018 ; 0.338	β	2.055	0.0438
1440	0.179	0.04 ; 0.4	β	2.304	0.0243

Estimated parameters (α and β), every 10 *min*.

Figure S4. Influence of conditioned individuals, related to section collective choice and consensus.

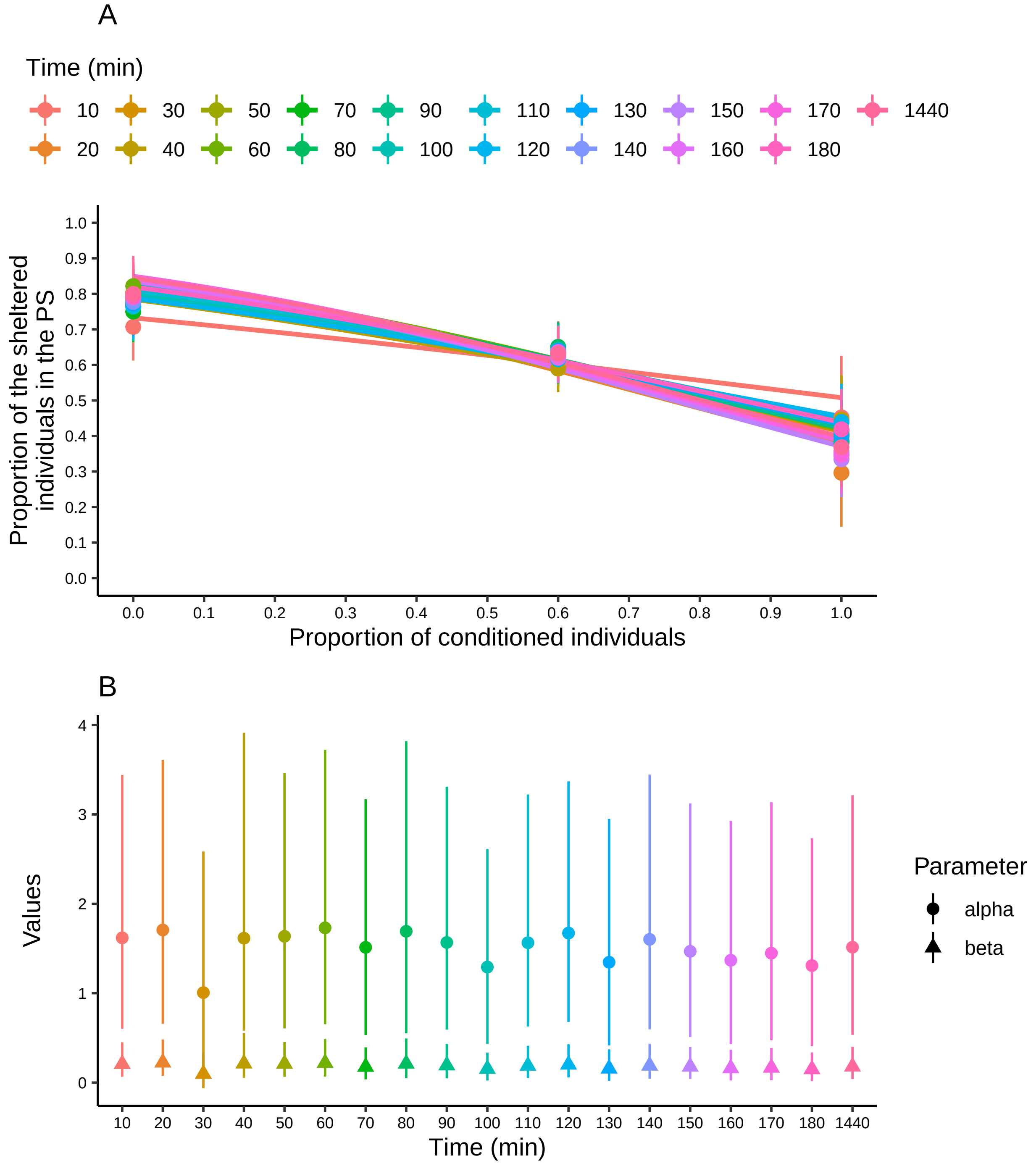

Influence of the proportion of conditioned individuals. A) Proportion of the sheltered population in the PS as a function of the proportion of the conditioned individuals and from *eq.* 1 of the main text, every 10 *minutes*. B) Estimated values of the parameters α (circle) and β (triangle) from *eq.* 1.

Figure S5. **Results of the resampling test, related to section Mechanism of shelter selection and model.**

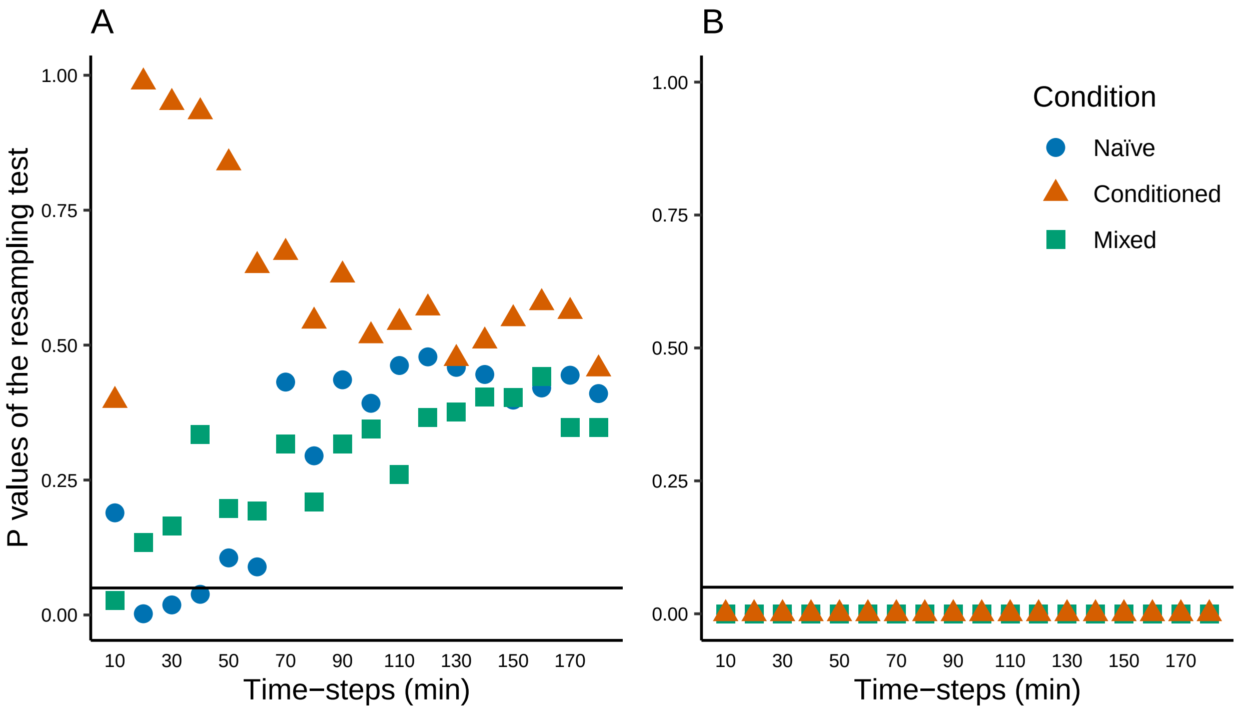

P values of the resampling test (10000 iterations) every 10 $minutes$, between the experimental and simulated results (from $eqs.$ $2-3$ of the main text). For the naïve (blue circle), for the conditioned (orange triangle) and for the mixed (green square) conditions. Solid horizontal line: significance value $\alpha = 0.05$. A) Unequal preference for the PS (parameter values of the naïve individuals: $\theta = 1\ 10^{-3}$; $\mu_{ps} = 0.63$, of the conditioned individuals: $\theta = 3.703\ 10^{-4}$; $\mu_{ps} = 0.445$). B) Equal preference for the PS (parameter values of the naïve individuals: $\theta = 1\ 10^{-3}$; $\mu_{ps} = 0.5$, of the conditioned individual: $\theta = 3.703\ 10^{-4}$; $\mu_{ps} = 0.5$). Other parameter values: $\rho = 0.002$; $\zeta = 1.19$.

Figure S6. Simulated sheltered population, related to section Mechanism of shelter selection and model.

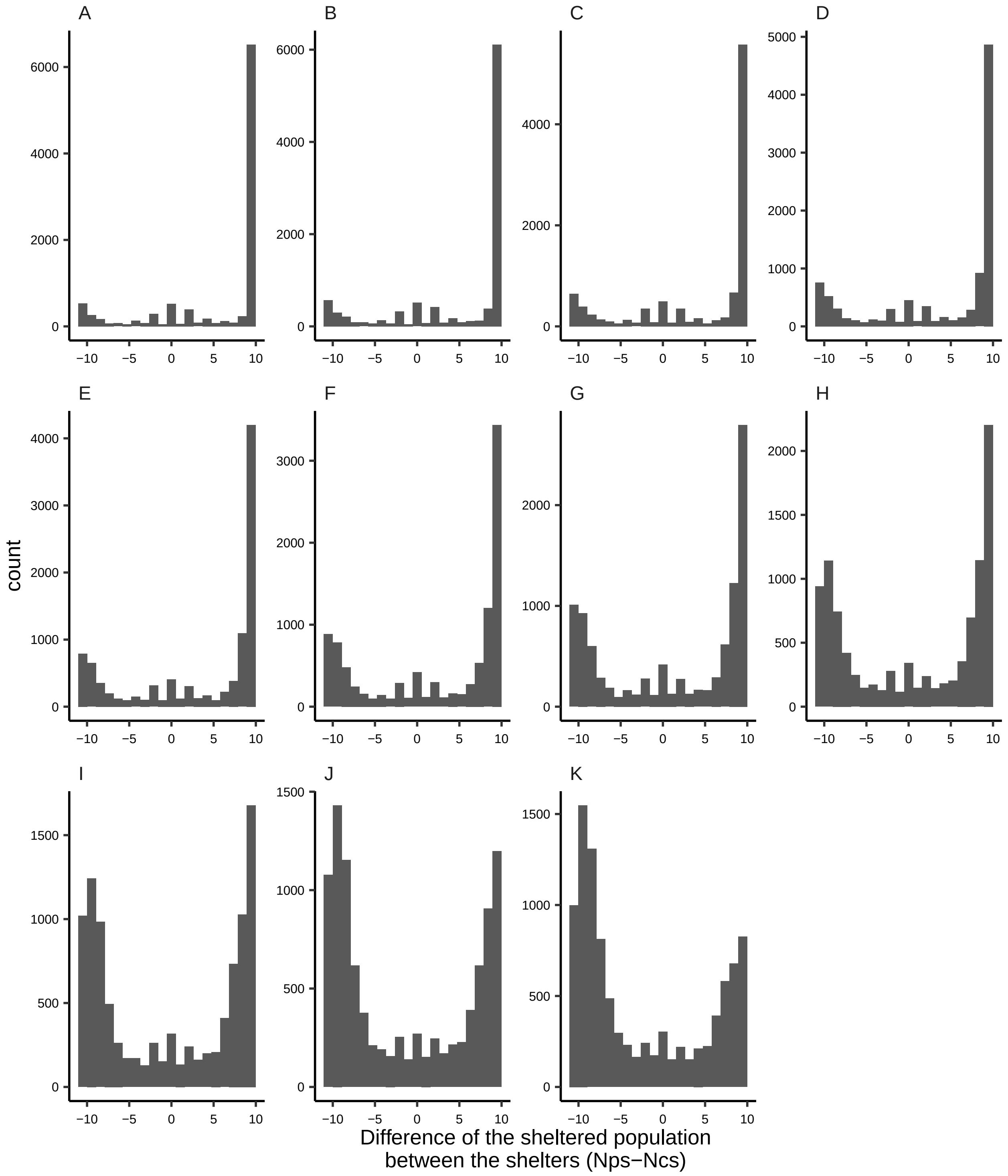

Distribution of the sheltered population difference between the PS and the CS ($N_{PS} - N_{CS}$) at 10800 *seconds*, from the stochastic simulations (10000 realisations from *eqs*. $2-3$, parameters values from table 2 of the main text). A-K) Different number of naïve individuals in mixed groups (Ngroup = 10 individuals): A) 10. B) 9. C) 8. D) 7. E) 6. F) 5. G) 4. H) 3. I) 2. J) 1. K) 0.

Figure S7. Simulated sheltered distribution of the 100 % naïve individuals, extension from figure 4B.

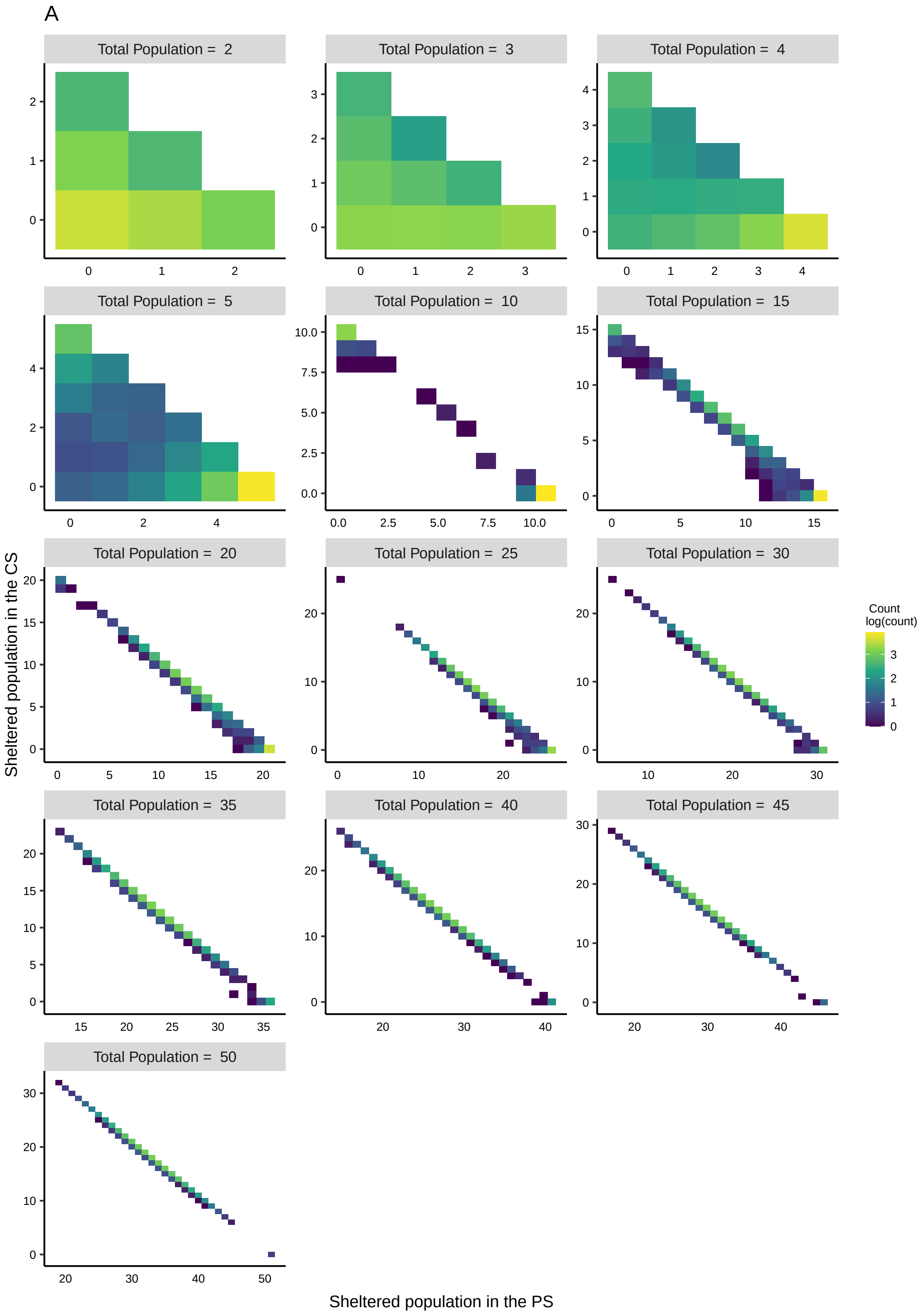

Two dimensional histograms of the simulated sheltered population (10000 iterations, from *eqs*. 2 − 3 of the main text) in the PS and the CS at 24 *hours* for population size of 2 to 50, For 100 % of naïve individuals.

Figure S8. Simulated sheltered distribution of mixed individuals (40 % naïve and 60 % conditioned individuals), extension from figure 4C.

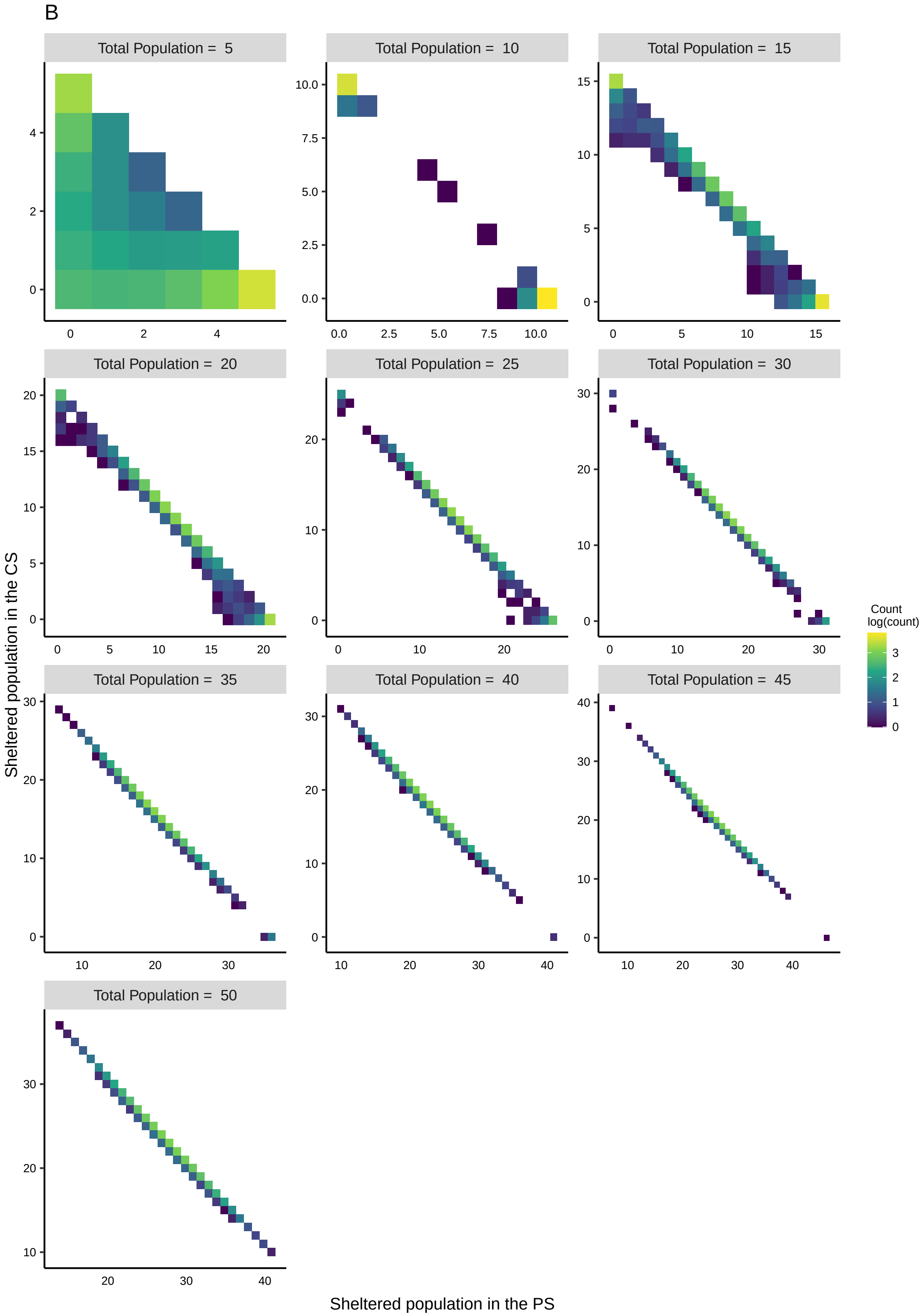

Two dimensional histograms of the simulated sheltered population (10000 iterations, from *eqs*. 2 − 3 of the main text) in the PS and the CS at 24 *hours* for population size of 2 to 50, For the mixed individuals (40 % naïve and 60 % conditioned individuals).

Figure S9. **Simulated sheltered distribution of the 100 % conditioned individuals, extension from figure 4D.**

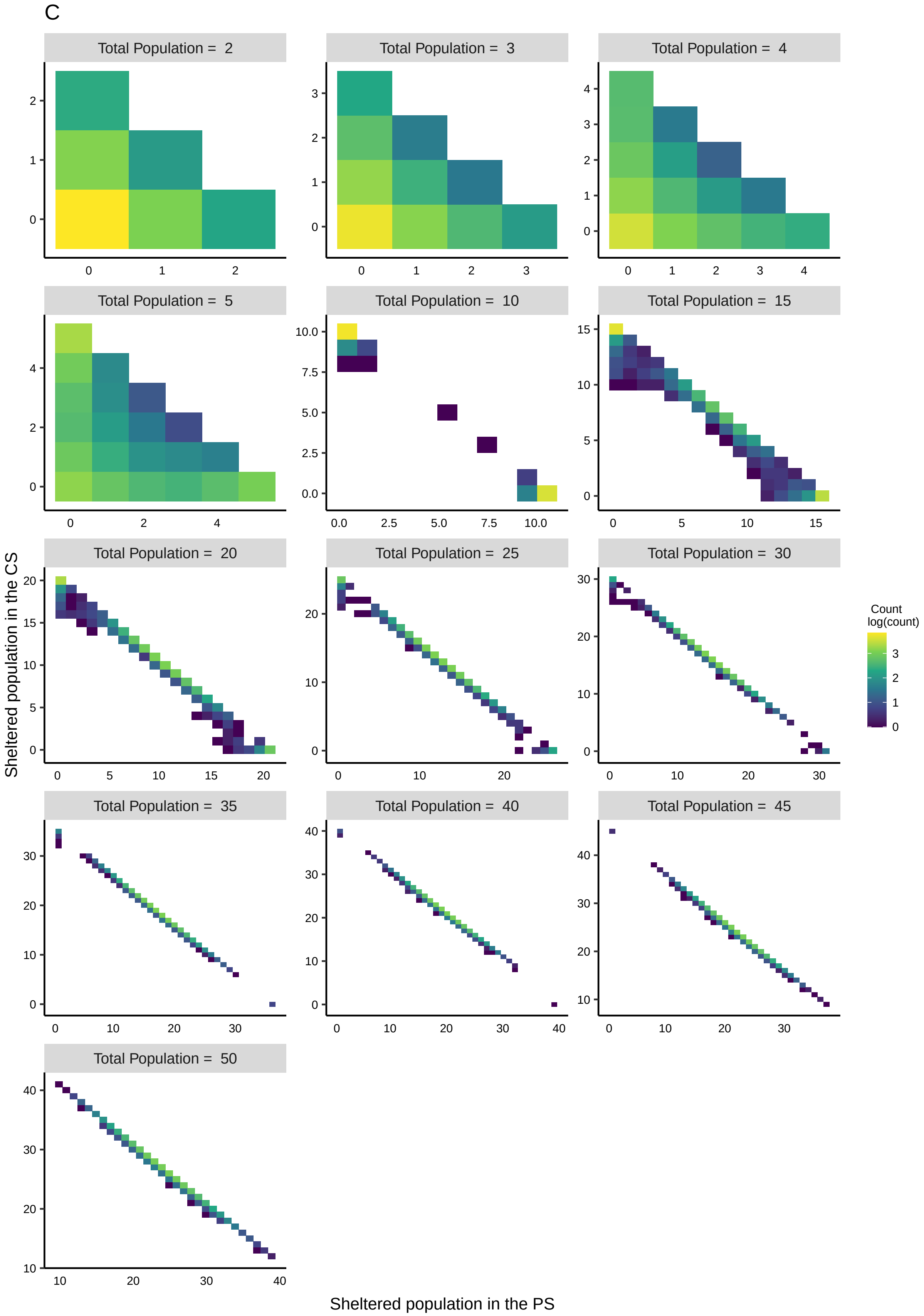

Two dimensional histograms of the simulated sheltered population (10000 iterations, from *eqs.* $2-3$ of the main text) in the PS and the CS at 24 *hours* for population size of 2 to 50, For 100 % of conditioned individuals.

Figure S10. **Conditioning device, related to STAR Methods**

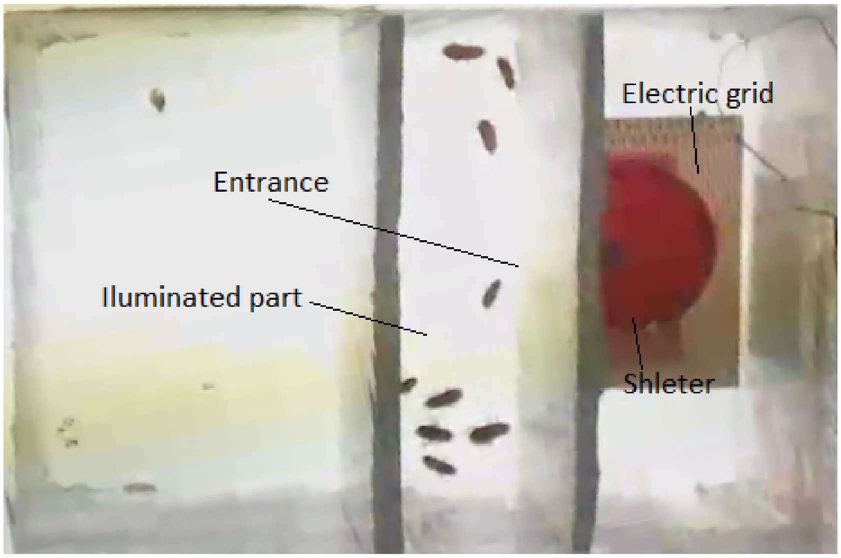

Figure S11. **Electric shocks administrated to each group, related to STAR Methods**

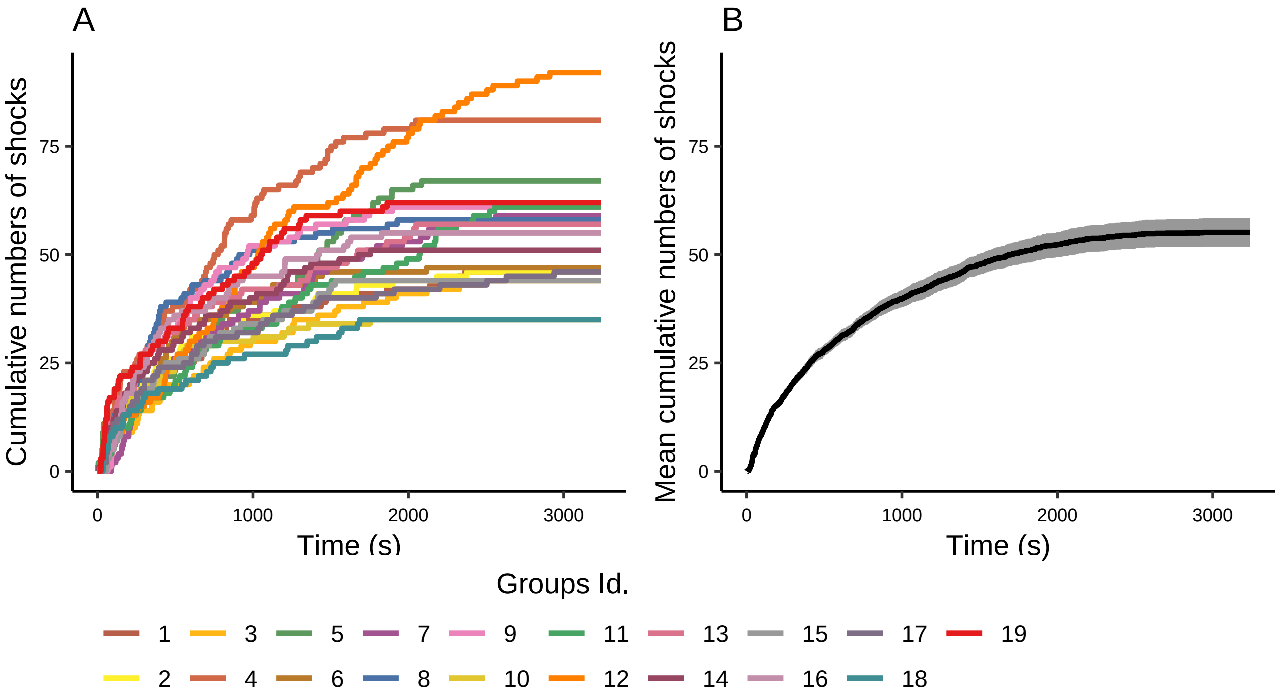

Electric shocks administrated over time (*seconds*). A) Cumulative numbers of electric shocks administrated to the groups of cockroaches (10 individuals) during the conditioning procedure for the conditioned condition. B) Mean $\pm$ SEM cumulative number of electric chocks over time.

Figure S12. **Experimental set-up, related to STAR Methods.**

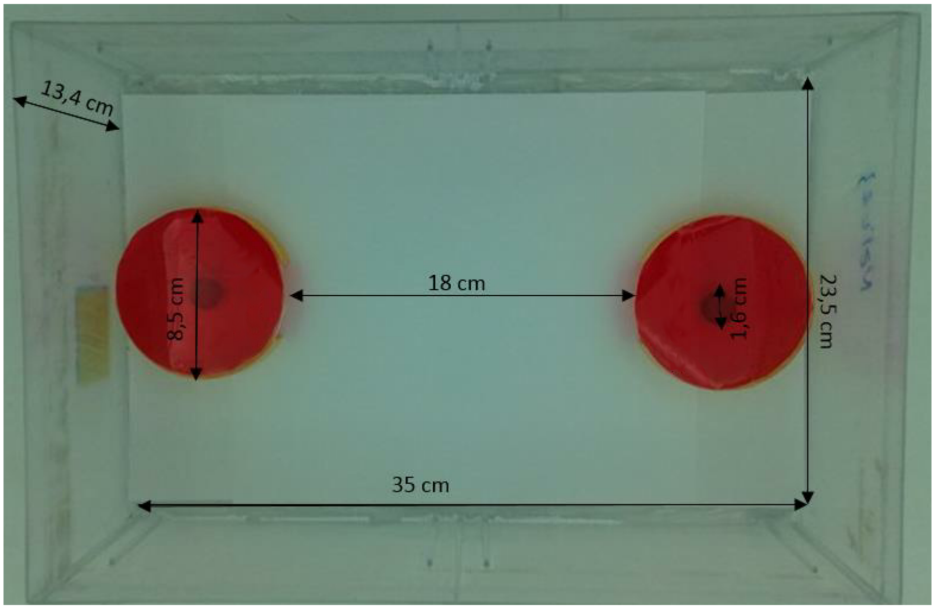

Supplement: Document S1. Figures S1–S12 and Tables S1–S3 [file mmc1.pdf]
